# Biotin-Linked Ursolic Acid Conjugates as Selective Anticancer Agents and Target-Identification Tools for Cancer Therapy

**DOI:** 10.3390/molecules30234588

**Published:** 2025-11-28

**Authors:** Riham M. Bokhtia, Kunj Bihari Gupta, Annabella Natalini, Theerth Vikas Srinivasan, Nihal Amineni, Sophia Ying, Rajeev Shakuja, Guido F. Verbeck, Bal L. Lokeshwar, Siva S. Panda

**Affiliations:** 1 Department of Pharmaceutical Organic Chemistry, Faculty of Pharmacy, Zagazig University, Zagazig 44519, Egypt; 2 Georgia Cancer Center, Augusta University, Augusta, GA 30912, USA; 3 Department of Chemistry and Biochemistry, Augusta University, Augusta, GA 30912, USA; 4 Department of Biomedical Engineering, Georgia Institute of Technology, Atlanta, GA 30332, USA; 5 Department of Chemistry, University of Delhi, New Delhi, Delhi 110007, India; 6 Department of Chemistry, Birla Institute of Technology and Science, Pilani, Rajasthan 333031, India; 7 Department of Biochemistry and Molecular Biology, Augusta University, Augusta, GA 30912, USA

**Keywords:** natural product, Ursolic acid, biotin, bioconjugate, microwave-assisted synthesis, in vitro assays, proteomics, anticancer agents, selectivity index

## Abstract

Ursolic acid (UA), a naturally occurring pentacyclic triterpenoid, exhibits potent anticancer properties; however, its poor solubility and bioavailability limit its therapeutic application. To overcome these challenges and facilitate molecular target identification, a set of biotin-conjugated UA derivatives (**5a**–**d**) was synthesized through selective C-28 alkylation and biotinylation. The use of microwave-assisted synthesis significantly enhanced both reaction efficiency and product purity. Among the derivatives, compound **5c** exhibited increased cytotoxicity and selectivity against bladder cancer cell lines, surpassing UA in its ability to induce apoptosis, generate reactive oxygen species (ROS), and halt cell cycle progression at the G1 phase. Proteomic profiling revealed that **5c** interacts with proteins involved in ER stress, RNA processing, cytoskeletal remodeling, and metabolic regulation. These findings underscore the potential of biotinylated UA derivatives as multifunctional chemical probes for mechanistic studies in the development of targeted therapies for cancer.

## 1. Introduction

**Figure 1:**
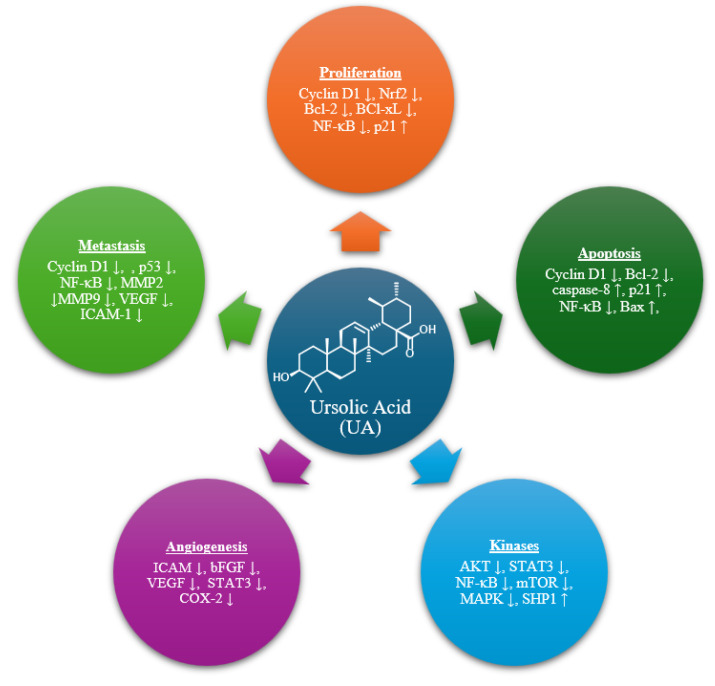
Molecular targets of UA.

Ursolic acid (UA) is a naturally occurring pentacyclic triterpenoid abundantly found in various medicinal plants and edible fruits [1,2,3,4,5]. It has garnered significant attention for its broad-spectrum pharmacological properties, particularly its activities against several cancers in experimental models. UA exhibits diverse anticancer mechanisms, including apoptosis, autophagy, inhibition of angiogenesis, and suppression of tumor growth across multiple cancer types [6]. Additionally, multiple preclinical studies have highlighted its potential to suppress tumor growth. Investigations into UA’s pharmacokinetics in human volunteers have also indicated that it possesses a favorable safety profile [7,8,9]. Furthermore, UA impedes metastasis and angiogenesis by modulating key molecular targets and signaling cascades such as PI3K/Akt, MAPK, NF-κB, and Wnt/β-catenin (Figure 1) [10,11].

Despite its promising therapeutic potential, the clinical application of UA is hindered by several pharmacokinetic limitations, including poor aqueous solubility, low oral bioavailability, and rapid systemic clearance [12,13]. These challenges underscore the need for innovative strategies to enhance the drug-like properties of UA and facilitate its clinical translation.

Medicinal chemistry efforts have mainly focused on structural modifications at the C-3 hydroxyl and C-28 carboxylic acid groups, which are key pharmacophores responsible for UA’s biological activity [14]. Such derivatizations have not only enhanced UA’s pharmacokinetic and pharmacodynamic profiles but also facilitated the development of molecular probes for mechanistic studies [15,16]. A crucial step in drug development is identifying molecular targets, which can be accomplished through a combination of computational and experimental methods. In silico target prediction tools enable rapid screening based on structural similarity and known ligand-target interactions [17,18,19], whereas high-throughput screening (HTS) platforms provide empirical validation but require substantial resources [20]. Although UA is well recognized as a potential anticancer agent, its mechanism of action and molecular target are still unclear. Because of this uncertainty, the further use of UA and its analogs remains limited.

To identify the molecular targets of potential compounds, various approaches have been utilized. Affinity-based proteomic techniques have emerged as powerful tools for target identification, particularly when compounds are conjugated with affinity tags, such as biotin [21,22,23]. Methods such as drug affinity responsive target stability (DARTS) [24], cellular thermal shift assay (CETSA) [25], and activity-based protein profiling (ABPP) have proven effective in identifying direct binding partners within complex biological matrices [26,27].

Among these, biotinylation of natural products has emerged as a promising strategy in cancer research, offering a versatile approach to enhance drug delivery, efficacy, and safety. This method exploits the extremely strong biotin–streptavidin interaction to selectively isolate compound–protein complexes from biological samples and then identify them using mass spectrometry [28,29]. Biotinylated probes are created by attaching biotin to the targeted compound, allowing for the specific capture of interacting proteins using streptavidin-coated beads. Major benefits of this approach include: (i) high specificity and sensitivity; (ii) compatibility with both native and in vitro systems; (iii) wide applicability across different compound types; and (iv) easy integration with quantitative proteomics techniques like Stable Isotope Labeling by Amino acids in Cell culture (SILAC).

In addition, biotin receptors are often overexpressed in cancer cells compared to normal cells. Biotin-conjugated natural bioactive compounds are used to exploit receptor-mediated endocytosis, a cellular uptake mechanism facilitated by biotin receptors that are frequently overexpressed on the surface of cancer cells [30,31,32]. This targeted delivery system improves the bioavailability of therapeutic agents by promoting their selective internalization into malignant cells, thereby minimizing off-target effects and reducing systemic toxicity.

In this study, we describe the rational design, synthesis, and biological evaluation of biotin-conjugated UA derivatives as a new strategy to overcome UA’s pharmacokinetic limitations and identify its molecular targets. These conjugates maintain the bioactive UA scaffold while attaching biotin via customized linkers at the C-28 position, enabling dual functions: improved cellular uptake via biotin receptor-mediated endocytosis and affinity-based proteomic profiling to identify targets. Combining structural optimization with chemoproteomic capabilities, this approach not only enhances UA’s anticancer activity and selectivity against bladder cancer cells but also offers insights into mechanisms involving ER stress, RNA processing, and cytoskeletal remodeling. This work establishes biotinylated UA derivatives as versatile chemical probes with strong potential for advancing targeted cancer therapy and precision drug development.

## 2. Results and Discussion

### 2.1. Chemistry

**Scheme 1:**
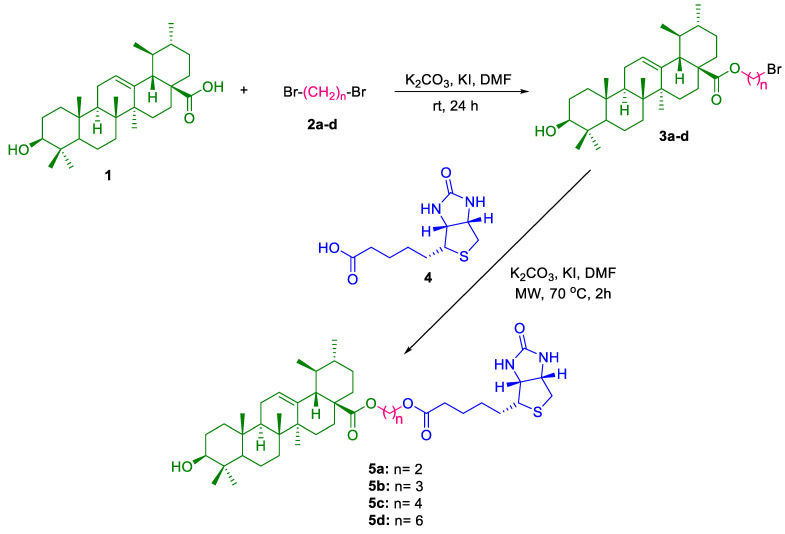
Synthesis of UA-biotin hybrid conjugates via alkyl linkers at C-28.

The synthetic route employed for the preparation of UA conjugates **5a**–**d** is illustrated in Scheme 1. The initial step involved the selective alkylation of the carboxylic acid moiety at the C-28 position of UA (**1**) using an excess of alkyl bromide in dry DMF, in the presence of potassium carbonate (K_2_CO_3_) and catalytic potassium iodide (KI) at ambient temperature. This reaction predominantly yielded monoalkylated derivatives (**3a**–**d**) as the major products, with minor formation of bis-alkylated byproducts. Spectroscopic analysis confirmed that alkylation occurred preferentially at the C-28 position, with no significant modification observed at the C-3 hydroxyl group under these conditions. However, when the reaction was conducted at elevated temperatures, partial O-alkylation at the C-3 hydroxyl group was detected, indicating temperature-dependent regioselectivity. The monoalkylated intermediates **3a**–**d** were subsequently purified by column chromatography and then conjugated with biotin (**4**). The final conjugates (**5a**–**d**) were synthesized using both conventional thermal methods and microwave-assisted synthesis. Notably, the microwave-assisted protocol significantly enhanced reaction efficiency, reducing the reaction time from 24 h to just 2 h while improving the yield and purity of the final products. All synthesized conjugates were thoroughly characterized using spectroscopic techniques such as IR, NMR, and HRMS, confirming their structural integrity and successful conjugation.

### 2.2. Antiproliferative Activity

**Figure 2:**
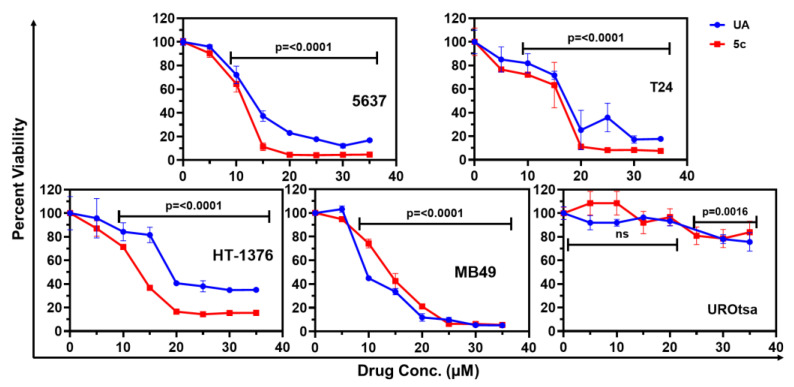
The percent cell viability of tumorigenic human (5637, T24, HT-1376), mouse (MB49), and non-tumorigenic bladder epithelial (UROtsa) cell lines after treatment with a selected dose of UA and **5c**. The percentage cell viability was calculated relative to the untreated control cells. MTT reduction assay was performed after 72 h of respective treatment. Results are expressed as the mean of percent cell viability ± standard deviation (SD) (n = 4), and one-way ANOVA was performed to determine the significance.

The synthesized hybrid conjugates **5a**–**d** were evaluated for their antiproliferative activity against the established human bladder cancer cell lines T24 and 5637. Test compounds were dissolved in ultrapure dimethyl sulfoxide (DMSO), diluted in cell culture media, added to cells cultured in 96-well cluster wells, and incubated for 72 h. The concentration of DMSO in culture media was maintained at < 0.05%. As previously reported, the fraction of viable cells in the culture wells was compared to that in cultures with 0.05% DMSO-only using a colorimetric assay, the Methyl Tetrazolium Bromide (MTT) reduction assay [16]. We determined the selectivity index (SI) of the compounds **5a**–**d** against tumor cells by comparing their cytotoxicity to that of a non-tumorigenic breast epithelial cell line, MCF-12A, as well as a non-tumorigenic, immortalized urothelial cell line, UROtsa (Table 1, and Figure 2) [33].

The MTT assay is frequently used in high-throughput screening for anticancer compounds due to its high sensitivity, ability to provide quantitative results, ease of use, affordability, compatibility with high-throughput screening equipment, and versatility across various cell types and experimental setups. It detects subtle changes in cell viability or proliferation. More importantly, this assay has served as a standard screening method for the antineoplastic compound library against a panel of 64 cancer cell lines, as determined by the National Cancer Institute.

The SI measures a cancer drug’s effectiveness in targeting tumor cells compared to non-tumorigenic cells. It is calculated by comparing the dose of the drug required to inhibit cancer cell growth to that required to inhibit the proliferation of non-tumorigenic cells. A higher SI indicates greater potency against cancer cells, whereas a lower SI may signal potential toxicity. The SI is crucial in guiding drug development and clinical decision-making to improve cancer treatment outcomes.

Compound **5c** showed promising activity against bladder cancer cells, with IC_50_ values of 14.20 µM for T24 cells and 10.97 µM for 5637 cells, and selectivity indices (SI) of 5.85 and 7.58, respectively. Compound **5a** demonstrated the most selective antiproliferative effects, with an IC_50_ of 14.57 µM for T24 and 39.31 µM for 5637, yielding high SIs of 42.96 and 15.92. In contrast, compound **5b** was ineffective, with IC_50_ values of 10,000 µM or higher for 5637 and 107.7 µM for T24, indicating minimal therapeutic potential. Similarly, compound **5d** was largely inactive. The parent compound, UA, exhibited moderate cytotoxicity, with IC50 values of 13.43 µM for 5637 and 17.52 µM for T24, resulting in SIs of 4.19 and 3.21, respectively, indicating a modest therapeutic window but less selectivity than compounds **5a** and **5c**.

The biological data (Table 1) show that linker length significantly influences cytotoxicity and selectivity. Among the synthesized conjugates, **5c**, which has a four-carbon linker, demonstrated the best balance of potency and selectivity across bladder cancer cell lines. In contrast, shorter (two-carbon, **5a**) or longer linkers (**5d**) resulted in decreased activity or loss of selectivity, while **5b** (three-carbon) exhibited minimal cytotoxicity. These findings suggest that linker length and flexibility can affect the orientation of biotin relative to the UA scaffold, impacting receptor-mediated uptake and access to intracellular targets. The optimal linker length likely enables effective interaction with biotin receptors and maintains UA’s pharmacophoric interactions, whereas too short or too long linkers may hinder these processes by limiting conformational flexibility or causing steric hindrance. This Structure–Activity Relationship (SAR) insight highlights the importance of linker optimization in designing biotinylated UA derivatives for targeted cancer therapy.

As **5c** was identified as the most potent among the four synthesized biotinylated conjugates, it was further investigated for its cytotoxicity against additional bladder cancer cell lines, such as MB49, a highly aggressive murine bladder cancer cell line mimicking a phenotype of muscle-invasive bladder cancers, and HT-1376, a human high-grade bladder cancer cell line. The IC_50_ values indicate that **5c** is effective across multiple bladder cancer cell lines (Figure 2 and Table 2).

### 2.3. Induction of Reactive Oxygen Species by UA and Compound **5c** in Bladder Cancer Cells

**Figure 3:**
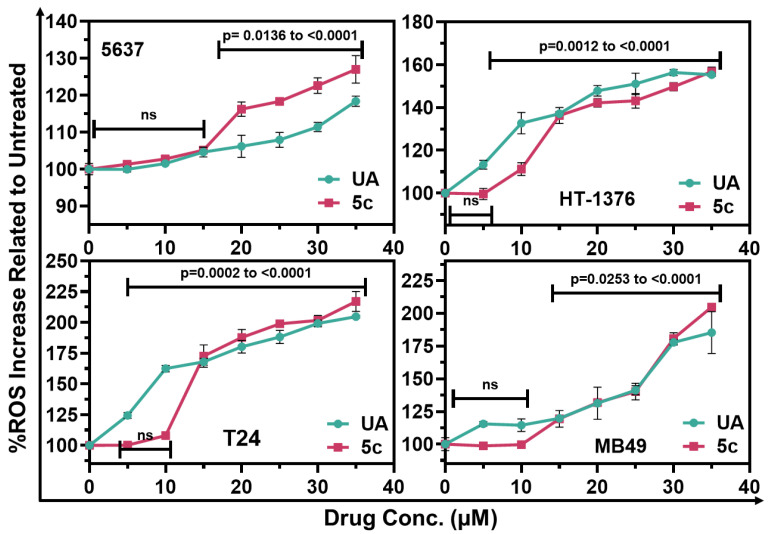
Estimation of oxidative stress: Treatment with UA or **5c** increased ROS levels in various bladder cancer cells. ROS levels were estimated at 24 h using DCFDA. The relative fluorescence unit (RFU) was normalized to that observed in untreated cells and expressed as a percent increase. Data are Means ± SD, (n = 4). Relative significance, expressed as *p*-value are calculated based on one-way ANOVA.

Since **5c** was not toxic to non-tumorigenic cells, such as MCF12A and UROtsa, these cells were excluded from experiments aimed at identifying the broad mechanism of cytotoxicity. A common mechanism by which these antioxidants induce cytotoxicity in tumor cells is by generating Reactive Oxygen Species (ROS) selectively in fast-growing tumor cells. The levels of ROS generated by **5c** exposed cells were evaluated with the oxidation of 2′,7′-dichlorodihydrofluorescein diacetate (H2DCFDA) [34]. It is a cell-permeant fluorescent probe used for detecting ROS production (Thermo Scientific Inc., Waltham, MA, USA). As shown in Figure 3, we observed a significant increase in ROS production when cells were exposed to a range of drug doses (5–40 µM). Figure 3 illustrates the quantification of intracellular ROS levels in four bladder cancer cell lines, 5637, T24, HT-1376, and MB49, following treatment with UA and its biotin-conjugated derivative, compound **5c**.

The human bladder cancer cells from 5637 and HT1376 exhibited a consistent trend where compound **5c** generated higher levels of ROS than UA, especially at concentrations above 20 µM. This variation in response may suggest increased cellular uptake or better mitochondrial targeting, linked to the biotin-conjugated derivative, as demonstrated by the Wang research group in their study on targeted drug delivery using biotin conjugates [35]. In T24 cells, both compounds induced comparable increases in ROS levels across all tested concentrations, suggesting that the biotin modification did not significantly impact the ROS-inducing capacity in these cells, further substantiating the hypothesis that biotin conjugation can enhance pro-oxidant activity in particular cellular contexts. In MB49 cells, both UA and compound **5c** resulted in a concentration-dependent increase in ROS levels. Notably, compound **5c** elicited a more robust ROS response at elevated concentrations (30–40 µM), indicating enhanced oxidative stress induction relative to UA.

These findings indicate that compound **5c** increases ROS generation in bladder cancer cells, potentially contributing to its cytotoxic effects. The varied ROS profiles among different cell lines underscore the significance of cellular context in determining therapeutic efficacy.

### 2.4. Apoptosis Induction by UA and **5c** in Bladder Cancer Cells

**Figure 4:**
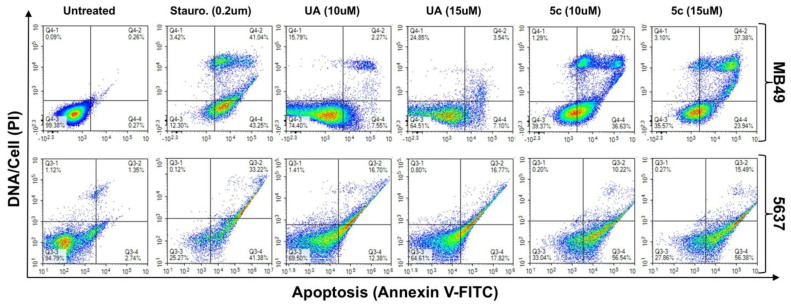
Estimating apoptotic cell death: Representative quadrant analysis images of bladder cancer cells 5637 and MB49 treated for 24 h with UA or **5c** (10 & 15 μM each), or Staurosporine (Sta, 0.2 μM; a positive inducer of apoptosis). The intensity of Annexin V-FITC (bound to externalized phosphatidyl serine) and PI (DNA) in a single cell suspension was measured using the NovoCyte Quanteon flow-cytometer. The intensity of green and red fluorescence were recorded, and quadrant analysis was performed using NovoExpress software (version 1.6.1).

**Figure 5:**
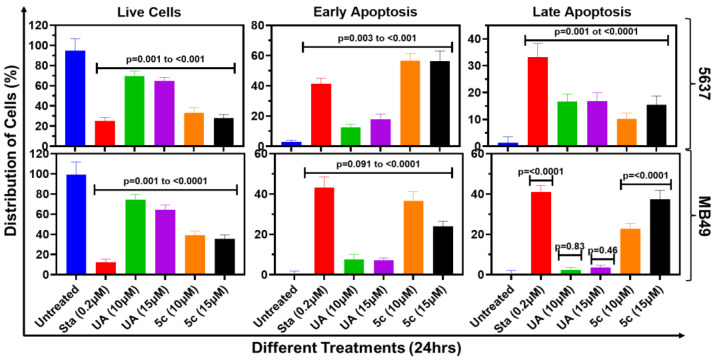
Quantitative representation of apoptotic cell death: The absolute quantification of early and late apoptosis in 5637 & MB49 cells is presented as bar graphs. Data shown are mean ± SD of three experiments (n = 3), and one-way ANOVA was performed to determine the significance of the differences between untreated control and treated cell samples.

The potential of cells treated with UA or **5c** to undergo dose-dependent apoptosis was explored using quantitative flow cytometric analysis, as shown in Figure 4. Utilizing a kit that detects quantitative binding of Annexin V to externalized phosphatidyl serine during the apoptosis cascade and permeability of Propidium iodide (PI) in cells undergoing cell death, we determined the cells at various stages of cell death following 24h incubation with UA and **5c** in various bladder cancer cells. As shown in Figure 4, the data reveal a notable shift from viable cells (Q3) to apoptotic (Q2 and Q4) and potentially necrotic (Q1) populations, in treated cells in a dose-dependent manner. The untreated MB49 cells showed 99% viability, whereas treatment with 15 µM **5c** resulted in cells undergoing late apoptosis (Q2: 20%) and necrosis (Q1: 37%), ultimately reducing viability to ≤40%. A comparable trend was also noted in 5637 cells (Figure 5).

### 2.5. Cell-Cycle Analysis of Bladder Cancer Cells Treated with **5c**

**Figure 6:**
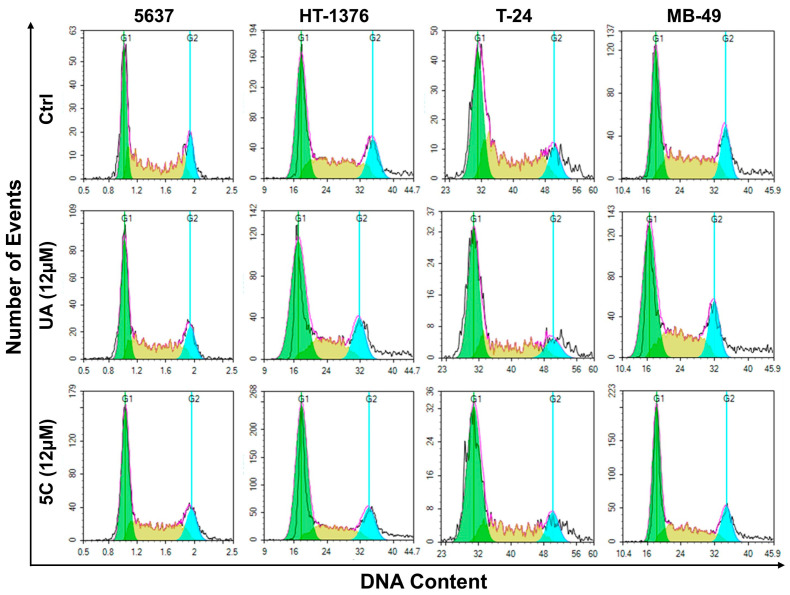
The representation of cell cycle phase distribution after treatment of UA and **5c** in the different bladder cancer cell lines (5637, HT-1376, T24, MB49). The flow cytometric images shown here are from the 3 independent experiments of data acquisition using NovoCyte Quanteon flow cytometer and NovoExpress software (version 1.6.1).

Cells treated with some antiproliferative drugs are blocked from orderly progression from one stage of their mitotic cycle to the next due to inhibition of a specific stage of the cell cycle. Analysis by flow cytometry (FC) of DNA content in the nuclei of drug-treated cells is a well-established technique for evaluating the fractions of cells in each stage of the cell cycle based on their DNA content. In this study, we employed FC to examine whether treatment with UA or **5c** affects the orderly cell cycle progression from G0/G1, S, and G2M before entering Mitosis (M). Figure 6 represents the effect of UA and **5c** on cell cycle progression in four bladder cancer cell lines, 5637, HT-1376, T-24, and MB-49, after a 48h treatment at a concentration of 12 µM. Following the treatment, nuclei suspensions from treated cells were prepared by direct cell lysis in a hypotonic solution, with simultaneous staining with PI (100 µg/mL). The resulting nuclei suspension and the samples were run on the flow cytometer and analyzed for DNA content. Data collected were analyzed, and their distribution across the G1, S, and G2/M phases is displayed in Figure 6 as histograms.

The histograms displayed distinct alterations in cell cycle profiles post-treatment. Compared to the untreated controls, **5c** treatment resulted in a marked accumulation of cells in the G1 phase in most cell lines, particularly in 5637 and HT-1376, suggesting a G1 phase arrest. In contrast, the cells treated with UA showed a more modest shift in phase distribution. Notably, T-24 and MB-49 cells also exhibited increased populations in G1 with **5c** treatment, accompanied by a reduction in S-phase cells, indicating impaired DNA synthesis. These findings imply that compound **5c** exerts its antiproliferative effects, at least in part, by inducing G1 phase arrest, thereby inhibiting cell cycle progression in bladder cancer cells.

**Figure 7:**
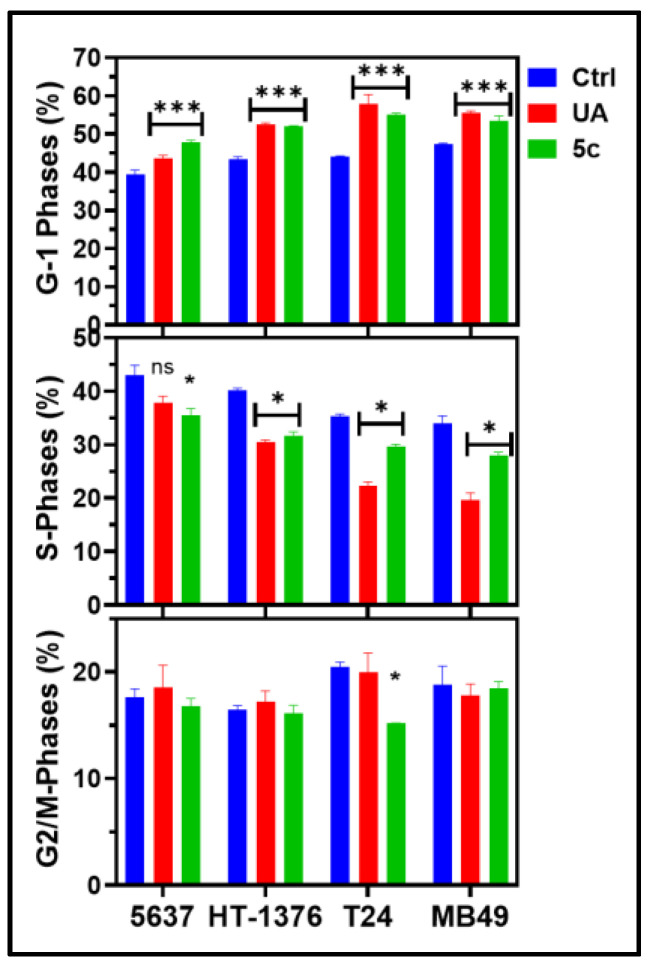
UA & **5c** induced cytotoxicity reveals cell cycle arrest in G1 phase: Bar graph representing the different phases of the cell cycle. The phases of the cell cycle resolution were analyzed using NovoCyte Quanteon flow cytometry in the 5637, HT-1376, T24, and MB49 cells after 24 h of treatment with UA and **5c**. The data were analyzed using NovoExpress software (version 1.6.1). The data shown here are the mean ± SD of n = 3, and a one-way ANOVA was performed to assess significance (* *p* > 0.05; *** *p* > 0.001; ns = Statistically non-significant).

Treatment with **5c** led to a significant alteration in the cell cycle distribution; a consistent and greater accumulation of cells in the G1 phase, accompanied by a corresponding reduction in the S and G2/M phases, indicating a G1-phase arrest. This finding suggests that the compounds interfere with the transition from G1 to S phase, thereby inhibiting DNA synthesis and subsequent progression into mitosis (Figure 7, Table S1). Compared with the untreated control, treatment with compound **5c** resulted in approximately 20–30% increases in the G1-phase population across the tested cell lines, with the most pronounced effects observed in 5637 and HT-1376 cells. This shift was paralleled by a significant decrease in the S phase, reflecting impaired DNA replication. These alterations in cell cycle distribution were statistically significant and support the conclusion that the antiproliferative activity of compound **5c** is, at least in part, mediated through cell cycle arrest at the G1 checkpoint.

## 3. Proteomics to Identify Protein Targets of 5c in Bladder Cancer Cells

We used proteomics to identify potential molecular targets of **5c**. A pulldown assay with streptavidin-agarose-coated magnetic beads was performed. Briefly, 5637 and MB49 cells were incubated with 50 µM **5c** for 10 min and 1 h. After incubation, the cells were rinsed three times with cold phosphate-buffered saline (PBS), and cell lysates were prepared and clarified by centrifugation to remove cellular debris, including nuclei. The clarified lysates were incubated with streptavidin-coated magnetic beads to selectively bind biotinylated contents. Bound proteins were separated from unbound proteins through thorough bead washing. Proteins attached to the beads were eluted and analyzed on an Orbitrap Fusion Tribrid mass spectrometer (Thermo Scientific, New York, NY, USA) coupled with an Ultimate 3000 nano-UPLC system. Samples were trapped on a PepMap100 C18 trap column (5 µm, 0.3 × 5 mm) and separated on a PepMap RSLC C18 column (2 µm, 75 µm × 150 mm) using a gradient from 2 to 40% acetonitrile with 0.1% formic acid over 120 min at 300 mL/min and 40 °C. MS data were acquired in positive mode using data-dependent acquisition (DDA), with precursor scans at 120,000 FWHM (400–2000 *m*/*z*), and MS/MS scans in top-speed mode (3 s cycle) using HCD fragmentation (NCE 32%). Proteins were identified and quantified based on peptide spectral matches (PSMs), and fold enrichment compared to controls was calculated to evaluate interaction strength. The results showed a broad spectrum of enriched proteins, indicating that 5c interacts with both direct and indirect protein partners, providing insights into its potential mechanism of action.

The time-dependent binding of cellular proteins to **5c** in 5637 and MB49 cell lysates, analyzed by proteomics, revealed striking differences in protein abundance dynamics following treatment, underscoring distinct adaptive strategies employed by these cells (Table 3). The fold-change patterns at 10 min and 1h post-treatment compared to control highlight key pathways involved in energy metabolism, translational regulation, stress response, and chromatin remodeling.

### 3.1. Mitochondrial Bioenergetics and ATP Synthase Complex

Both cell lines exhibited upregulation of ATP synthase subunits, indicating enhanced mitochondrial activity [36]. In 5637 cells, ATP synthase subunit β showed a dramatic increase (3.29-fold at 10 min, 5-fold at 1 h), while MB49 cells displayed a modest rise (1.31-fold and 1.82-fold). Subunit O was consistently elevated in both lines, but MB49 cells showed a stronger response (4-fold at 10 min and 5-fold at 1 h). These findings suggest that MB49 cells may rely on rapid mitochondrial activation, whereas 5637 cells exhibit sustained metabolic reprogramming.

### 3.2. Translation Machinery and Elongation Factors

Translational control emerged as a major adaptive mechanism, particularly in 5637 cells [37]. eEF2 showed an extraordinary increase (44-fold at 10 min, 50-fold at 1 h), accompanied by initiation factors eIF4A (22-fold, 19-fold) and eIF5B (32-fold, 34-fold). MB49 cells exhibited only moderate changes (eEF2: 3-fold at 10 min, 2-fold at 1 h), suggesting a less aggressive translational response. The surge in elongation factors EF1β and EF1γ in MB49 cells (5-fold at 10 min) indicates an early but transient activation of protein synthesis.

### 3.3. Stress Response and Chaperone Networks

Heat shock proteins were among the most differentially regulated proteins [38]. In 5637 cells, HSP90 isoforms showed extreme induction (HSP90α: 119-fold; HSP90β: 93-fold at 10 min), sustained at 1 h, reflecting a robust proteostasis mechanism. MB49 cells displayed minimal changes (1.07–1.71-fold), suggesting a lower reliance on chaperone-mediated stabilization under stress.

### 3.4. RNA Processing and Splicing Factors

Splicing regulators such as U2AF65 and PUF60 were significantly upregulated in both cell lines, but the magnitude was higher in 5637 (U2AF65: 14-fold at 10 min, 10-fold at 1 h) compared to MB49 (4-fold and 3-fold). This indicates accelerated transcriptome remodeling in 5637 cells, potentially supporting rapid adaptation and survival [39].

### 3.5. Cytoskeletal Remodeling

Filamin-A (FLNA) showed consistent elevation in both lines, with 5637 cells exhibiting a stronger response (16-fold at 10 min, 18-fold at 1 h) compared to MB49 (3.33-fold and 4-fold). This suggests enhanced cytoskeletal reorganization in 5637, possibly linked to increased motility and invasiveness [40].

### 3.6. Chromatin Dynamics and Histone Variants

Histone proteins displayed marked differences between the two cell lines. 5637 cells showed substantial enrichment of histone H4 (13.23-fold at 10 min, 11.08-fold at 1 h) and H2A.Z (5.85-fold, 4.78-fold), indicating extensive chromatin remodeling. MB49 cells exhibited only modest changes (H4: 1.64-fold, 1.18-fold), suggesting a less pronounced epigenetic response [41].

Collectively, these data reveal that 5637 cells engage a highly aggressive and sustained adaptive program, characterized by massive upregulation of translational machinery, chaperone networks, and chromatin modifiers. In contrast, MB49 cells exhibit a more moderate and transient response, relying primarily on mitochondrial activation and limited translational enhancement. These differences may reflect intrinsic variations in tumor aggressiveness and could inform therapeutic strategies targeting protein synthesis and stress-response pathways.

## 4. Conclusions

This study demonstrates a rational approach to enhancing the therapeutic potential of UA via biotin conjugation, enabling improved selectivity and mechanistic investigation in bladder cancer models. We successfully synthesized and characterized a series of biotin-linked UA derivatives and assessed their biological activity. Among these, compound **5c** showed the most promising profile, exhibiting strong antiproliferative effects, selective cytotoxicity, and induction of apoptosis and ROS in multiple bladder cancer cell lines. Flow cytometry confirmed G1-phase cell cycle arrest as a key mechanism of growth inhibition. Importantly, affinity-based proteomic profiling revealed interactions with proteins involved in ER stress, RNA processing, cytoskeletal remodeling, and metabolic regulation, providing new insights into UA’s mode of action.

While these findings highlight the usefulness of biotinylated UA derivatives as versatile chemical probes for both therapeutic purposes and target discovery, future efforts will focus on confirming the identified protein targets, improving linker chemistry by exploring various linkers with different polarities, and evaluating the in vivo efficacy and pharmacokinetics of **5c**, paving the way for its development as a targeted anticancer agent and chemoproteomic tool.

## 5. Experimental Section

All chemicals were obtained from Fisher Scientific (Waltham, MA, USA) and MilliporeSigma (St. Louis, MO, USA) and used as received without further purification. Thin-layer chromatography (TLC) was employed to monitor reaction progress and assess compound purity, using a solvent system of ethyl acetate (99.0%, Fisher Scientific, Waltham, MA, USA) and n-hexane (99.0%, Fisher Scientific, Waltham, MA, USA) in a 3:7 ratio. TLC plates were pre-coated silica gel 60 F254 (MilliporeSigma, St. Louis, MO, USA), and spots were visualized under UV light at 254 nm or by exposure to iodine vapors. Retardation factor (Rf) values ranged between 0.50 and 0.70. Microwave-assisted reactions were performed in a single-mode cavity Discover Microwave Synthesizer (CEM Corporation, NC, USA). Melting points were determined using a capillary melting point apparatus equipped with a digital thermometer (Stanford Research Systems, Sunnyvale, CA, USA). Nuclear Magnetic Resonance (NMR) spectra were recorded in CDCl_3_ on a Bruker spectrometer operating at 500 MHz for ^1^H and 125 MHz for ^13^C, with tetramethylsilane (TMS) as an internal standard. Analyses were conducted at the NMR facility in the Department of Chemistry and Biochemistry, Augusta University (Augusta, GA, USA). Infrared (IR) spectra (KBr pellets, cm^−1^) were obtained using a Nicolet iS5 spectrophotometer (Thermo Fisher Scientific, Waltham, MA, USA). Mass spectrometric data were collected on an Agilent Technologies 6545 Q-TOF LC/MS system (Agilent Technologies, Inc., Santa Clara, CA, USA). High-performance liquid chromatography (HPLC) analyses were performed using an Agilent 1100 Series system coupled with a 1260 Infinity II LC module (Agilent Technologies, Inc., Santa Clara, CA, USA).

### 5.1. General Method for Preparation of Compounds **3a**–**d** [16]

UA **1** (500 mg, 1.0 equiv.) and K_2_CO_3_ (303 mg, 2.0 equiv.) were added to DMF (15 mL) and stirred at room temperature for 30 min. Then, dibromoalkane **2a**–**d** (4.0 equiv.) and KI (91 mg, 0.5 equiv.) were added to the mixture. The reaction mixture was stirred for 24 h at room temperature, and its progress was monitored by TLC. After completion of the reaction, the mixture was poured into 100 mL of cold distilled water and partitioned with ethyl acetate (20 mL) three times. The organic layer was washed with brine, dried over sodium sulfate, and purified by column chromatography (5% ethyl acetate/hexanes) to obtain compounds **3a**–**d**.

(2-Bromoethyl) 3-hydroxy-urs-12-en-28-oate (**3a**)

White powder, m.p. 132–134 °C, yield: 84% (518 mg). IR: *ν*_max_/cm^−1^; 3420, 2922, 2868, 1720, 1453, 1139; ^1^HNMR (CDCl_3_) δ: 5.25 (t, *J* = 3.6 Hz, 1H, CH), 4.28 (t, *J* = 6.1 Hz, 2H, CH_2_), 4.10 (q, *J* = 7.1 Hz, 1H, CH), 3.46 (t, *J* = 6.1 Hz, 2H, CH_2_), 3.19 (dd, *J* = 11.1, 4.9 Hz, 1H, CH), 2.21 (d, *J* = 11.1 Hz, 1H, CH), 2.02–1.95 (m, 3H, (1H, CH) + (2H, CH_2_)), 1.88 (dd, *J* = 8.9, 3.6 Hz, 2H, CH_2_), 1.77 (td, *J* = 13.7, 4.6 Hz, 1H, CH), 1.69–1.44 (m, 9H, (1H, CH) + (8H, 4CH_2_)), 1.37–1.22 (m, 5H, (2H, CH_2_) + (3H, CH_3_)), 1.09–0.81 (m, 16H, (4H, 2CH_2_) + (12H, 4CH_3_)), 0.76 (s, 3H, CH_3_), 0.74 (s, 3H, CH_3_).

(3-Bromopropyl) 3-hydroxy-urs-12-en-28-oate (**3b**)

White powder, m.p. 142–144 °C, yield: 75% (474 mg). IR: *ν*_max_/cm^−1^; 3365, 2924, 2869, 1721, 1672, 1454, 1197; ^1^HNMR (CDCl_3_) δ: 5.21 (t, *J* = 3.6 Hz, 1H, CH), 4.16–4.04 (m, 2H, CH_2_), 3.44 (t, *J* = 6.6 Hz, 2H, CH_2_), 3.19 (dd, *J* = 11.1, 4.9 Hz, 1H, CH), 2.19 (d, *J* = 11.0 Hz, 1H, CH), 2.15–2.10 (m, 2H, CH_2_), 2.02–1.95 (m, 2H, CH_2_), 1.89 (dd, *J* = 8.8, 3.6 Hz, 2H, CH_2_), 1.76 (td, *J* = 13.7, 4.6 Hz, 1H, CH), 1.67–1.43 (m, 9H, (1H, CH) + (8H, 4CH_2_)), 1.37–1.22 (m, 5H, (1H, CH) + (4H, 2CH_2_)), 1.06–0.83 (m, 18H, (1H, CH) + (2H, CH_2_) + (15H, 5CH_3_)), 0.76 (s, 3H, CH_3_), 0.74 (s, 3H, CH_3_).

(4-Bromobutyl) 3-hydroxy-urs-12-en-28-oate (**3c**)

White solid, m.p. 156–158 °C, yield: 69% (447 mg). IR: *ν*_max_/cm^−1^; 3345, 2924, 2869, 1720, 1672, 1455, 1197; ^1^HNMR (CDCl_3_) δ: 5.21 (t, *J* = 3.6 Hz, 1H, CH), 4.09 (q, *J* = 7.1 Hz, 1H, CH), 4.05–3.94 (m, 2H, CH_2_), 3.40 (t, *J* = 6.7 Hz, 2H, CH_2_), 3.19 (dd, *J* = 11.1, 4.9 Hz, 1H, CH), 2.19 (d, *J* = 11.2 Hz, 1H, CH), 2.01–1.87 (m, 5H, (1H, CH) + (4H, 2CH_2_)), 1.77–1.71 (m, 3H, (1H, CH) + (2H, CH_2_)), 1.66–1.43 (m, 9H, (1H, CH) + (8H, 4CH_2_)), 1.37–1.22 (m, 5H, (2H, CH_2_) + (3H, CH_3_)), 1.05–0.89 (m, 15H, (6H, 3CH_2_) + (9H, 3CH_3_)), 0.83 (d, *J* = 6.5 Hz, 3H, CH_3_), 0.75 (s, 3H, CH_3_), 0.72 (s, 3H, CH_3_).

(6-Bromohexyl) 3-hydroxy-urs-12-en-28-oate (**3d**)

White solid, m.p. 168–170 °C, yield: 82% (557 mg). IR: *ν*_max_/cm^−1^; 3327, 2924, 2866, 1717, 1454, 1198; ^1^HNMR (CDCl_3_) δ: 5.21 (t, *J* = 3.6 Hz, 1H, CH), 4.01–3.92 (m, 2H, CH_2_), 3.38 (t, *J* = 6.8 Hz, 2H, CH_2_), 3.19 (dd, *J* = 11.1, 4.9 Hz, 1H, CH), 2.20 (d, *J* = 11.3 Hz, 1H, CH), 2.00–1.93 (m, 1H, CH), 1.89–1.82 (m, 4H, 2CH_2_), 1.75 (td, *J* = 13.7, 4.6 Hz, 1H, CH), 1.67–1.23 (m, 21H, (2H, 2CH) + (16H, 8CH_2_) + (3H, CH_3_)), 1.06–0.89 (m, 15H, (6H, 3CH_2_) + (9H, 3CH_3_)), 0.83 (d, *J* = 6.4 Hz, 3H, CH_3_), 0.76 (s, 3H, CH_3_), 0.73 (s, 3H, CH_3_).

### 5.2. General Method for the Preparation of Compounds **5a**-**d**

A dried, heavy-walled Pyrex reaction tube containing a small magnetic stir bar was charged with equimolar amounts (1.0 equiv.) of biotin **4** and compound **3a**–**d**. Additionally, K_2_CO_3_ (134 mg, 3.0 eq.) and KI (27 mg, 0.5 eq.) were added, dissolving them in DMF. The reaction mixture was subjected to microwave irradiation at 50 W and maintained at 70 °C for 2h. After completion, the mixture was allowed to cool below 30 °C using the built-in cooling system (approximately 10 min). The reaction was quenched by adding ice-cold water, and the resulting precipitate was collected by filtration. The crude solid was sequentially washed with 20 mL of a 10% aqueous sodium carbonate (Na_2_CO_3_) solution, followed by three washes with distilled water (each 20 mL). Final purification was achieved through recrystallization from aqueous ethanol, yielding the target compounds 5a–d in analytically pure form.

2-((5-((3aS,4S,6aR)-2-Oxohexahydro-1H-thieno [3,4-d] imidazol-4-yl) pentanoyl) oxy) ethyl 3-hydroxy-urs-12-en-28-oate (**5a**)

The crude product was purified using column chromatography (1% MeOH/DCM) to obtain white solid, **5a** in pure form, m.p. 120–122 °C, yield: 72%. IR: *ν*_max_/cm^−1^; 3240, 2923, 2869, 1699, 1455, 1197; ^1^HNMR (CDCl_3_) δ: 5.18 (s, 1H, CH), 4.46–4.44 (m, 1H, CH), 4.26–4.14 (m, 5H, (1H, CH) + (4H, 2CH_2_)), 3.16–3.10 (m, 2H, 2CH), 2.86–2.83 (m, 1H, CH), 2.69 (d, *J* = 12.7 Hz, 1H, CH), 2.29 (t, *J* = 7.3 Hz, 2H, CH_2_), 2.16 (d, *J* = 11.2 Hz, 1H, CH), 1.97–1.93 (m, 1H, CH), 1.85–1.84 (m, 1H, CH), 1.74–1.42 (m, 18H, 9CH_2_), 1.33–1.20 (m, 5H, (2H, CH_2_) + (3H, CH_3_)), 1.02–0.69 (m, 24H, (6H, 3CH_2_) + (18H, 6CH_3_)); ^13^CNMR (CDCl_3_) δ: 177.4, 173.5, 164.0, 138.1, 125.8, 79.0, 62.3, 62.1, 62.0, 60.3, 55.6, 55.3, 52.9, 48.3, 47.6, 42.2, 39.7, 39.1, 39.0, 38.9, 38.8, 37.1, 36.8, 33.9, 28.5, 28.4, 28.3, 28.1, 27.3, 24.9, 24.3, 23.7, 23.4, 21.3, 18.5, 17.2, 15.8, 15.6; HRMS: *m*/*z* for C_42_H_66_N_2_O_6_S [M + H]^+^ *Calcd.*: 727.4314, *Found*: 727.4129.

3-((5-((3aS,4S,6aR)-2-Oxohexahydro-1H-thieno [3,4-d] imidazol-4-yl) pentanoyl) oxy) propyl 3-hydroxy-urs-12-en-28-oate (**5b**)

White solid, m.p. 95–97 °C, yield: 94%. IR: *ν*_max_/cm^−1^; 3274, 2924, 1699, 1455, 1198; ^1^HNMR (CDCl_3_) δ: 5.21 (t, *J* = 3.2 Hz, 1H, CH), 4.54–4.52 (m, 1H, CH), 4.34–4.32 (m, 1H, CH), 4.16–3.99 (m, 4H, 2CH_2_), 3.21–3.14 (m, 2H, 2CH), 2.93–2.89 (m, 1H, CH), 2.75 (d, *J* = 12.9 Hz, 1H, CH), 2.31 (t, *J* = 7.4 Hz, 2H, CH_2_), 2.19 (d, *J* = 11.3 Hz, 1H, CH), 2.01–1.86 (m, 7H, (1H, CH) + (6H, 3CH_2_)), 1.73–1.43 (m, 15H, (1H, CH) + (14H, 7CH_2_)), 1.41–1.23 (m, 5H, (2H, CH_2_) + (3H, CH_3_)), 1.05–0.68 (m, 24H, (6H, 3CH_2_) + (18H, 6CH_3_)); ^13^CNMR (CDCl_3_) δ: 177.8, 173.7, 138.5, 125.8, 79.4, 62.7, 61.4, 60.9, 55.4, 53.1, 48.4, 47.7, 42.3, 39.8, 39.3, 39.1, 39.0, 38.8, 37.2, 37.0, 34.0, 30.9, 28.5, 28.4, 28.2, 24.9, 24.4, 23.8, 23.5, 21.4, 18.5, 17.3, 17.2, 15.9, 15.7; HRMS: *m*/*z* for C_43_H_68_N_2_O_6_S [M + H]^+^ *Calcd.*: 741.4471, *Found*: 741.4220.

4-((5-((3aS,4S,6aR)-2-Oxohexahydro-1H-thieno [3,4-d] imidazol-4-yl) pentanoyl) oxy) butyl 3-hydroxy-urs-12-en-28-oate (**5c**)

White solid, m.p. 97–99 °C, yield: 82%. IR: *ν*_max_/cm^−1^; 3252, 2924, 2869, 1699, 1455, 1197; ^1^HNMR (CDCl_3_) δ: 5.21 (t, *J* = 3.3 Hz, 1H, CH), 4.54–4.51 (m, 1H, CH), 4.32 (dd, *J* = 7.4, 4.6 Hz, 1H, CH), 4.08–3.95 (m, 4H, 2CH_2_), 3.21–3.14 (m, 2H, 2CH), 2.93–2.90 (m, 1H, CH), 2.75 (d, *J* = 12.8 Hz, 1H, CH), 2.31 (t, *J* = 7.4 Hz, 2H, CH_2_), 2.20 (d, *J* = 11.3 Hz, 1H, CH), 2.00–1.86 (m, 5H, (1H, CH) + (4H, 2CH_2_)), 1.72–1.46 (m, 19H, (1H, CH) + (18H, 9CH_2_)), 1.41–1.23 (m, 5H, (2H, CH_2_) + (3H, CH_3_)), 1.06–0.83 (m, 18H, (6H, 3CH_2_) + (12H, 4CH_3_)), 0.76 (s, 3H, CH_3_), 0.72 (s, 3H, CH_3_); ^13^CNMR (CDCl_3_) δ: 177.8, 173.8, 138.5, 125.8, 79.3, 64.3, 63.9, 55.4, 53.1, 48.3, 47.7, 42.3, 39.8, 39.3, 39.1, 39.0, 38.9, 37.2, 37.0, 30.9, 28.4, 28.2, 25.6, 25.5, 24.9, 24.5, 23.8, 23.5, 21.4, 18.5, 17.4, 17.2, 15.9, 15.7; HRMS: *m*/*z* for C_44_H_70_N_2_O_6_S [M + H]^+^ *Calcd.*: 755.4817, *Found*: 755.4365.

6-((5-((3aS,4S,6aR)-2-Oxohexahydro-1H-thieno [3,4-d] imidazol-4-yl) pentanoyl) oxy) hexyl 3-hydroxy-urs-12-en-28-oate (**5d**)

White solid, m.p. 94–96 °C, yield: 81%. IR: *ν*_max_/cm^−1^; 3263, 2925, 2866, 1699, 1455, 1198; ^1^HNMR (CDCl_3_) δ: 5.21 (t, *J* = 3.3 Hz, 1H, CH), 4.60–4.58 (m, 1H, CH), 4.38 (dd, *J* = 7.6, 4.6 Hz, 1H, CH), 4.04 (t, *J* = 6.7 Hz, 2H, CH_2_), 4.02–3.91 (m, 2H, CH_2_), 3.23–3.15 (m, 2H, 2CH), 2.94–2.91 (m, 1H, CH), 2.80 (d, *J* = 13.0 Hz, 1H, CH), 2.35–2.28 (m, 2H, CH_2_), 2.20 (d, *J* = 11.3 Hz, 1H, CH), 1.97 (td, *J* = 13.3, 4.4 Hz, 1H, CH), 1.91–1.84 (m, 2H, CH_2_), 1.79–1.44 (m, 20H, (1H, CH) + (16H, 8CH_2_) + (3H, CH_3_)), 1.36–1.26 (m, 8H, 4CH_2_), 1.06–0.69 (m, 26H, (8H, 4CH_2_) + (18H, 6CH_3_)); ^13^CNMR (CDCl_3_) δ: 207.2, 178.0, 173.9, 138.5, 125.7, 79.5, 64.7, 64.3, 55.4, 53.1, 48.3, 47.7, 42.3, 40.3, 39.8, 39.3, 39.1, 39.0, 38.8, 38.4, 37.2, 37.0, 34.0, 33.3, 31.1, 30.9, 28.8, 28.7, 28.5, 28.4, 28.2, 27.3, 26.0, 25.8, 24.9, 24.5, 23.8, 23.5, 21.4, 18.6, 17.4, 17.3, 16.0, 15.7; HRMS: *m*/*z* for C_46_H_74_N_2_O_6_S [M + H]^+^ *Calcd.*: 783.4340, *Found*: 783.4389.

### 5.3. Cell Culture and Maintenance

This study was conducted using three human tumorigenic bladder cancer cell lines (5637, HT-1376, T24), a murine bladder cancer cell line (MB49), and two normal human non-tumorigenic cell lines (UROtsa and MCF-12A). All cells were purchased from ATCC, USA, and maintained in the authors’ laboratory. All cells except MCF-12A were routinely cultured & sub-cultured (passaged) in complete medium [RPMI-1640 basal medium, with 10% fetal bovine serum, and gentamicin sulfate (10 mg/L)], as described previously [6]. MCF-12A cells were cultured and sub-cultured in DMEM+F12 media (1:1) with supplements [horse serum (5%), human epidermal growth factor (20 ng/mL), cholera toxin (10 ng/mL), bovine insulin (0.01 mg/mL), hydrocortisone (500 ng/mL), and gentamycin sulfate (10 mg/L)].

### 5.4. Testing the Compounds for Antiproliferative Activity

The antiproliferative activities of various compounds examined in this report were measured using the MTT (3-[4,5-dimethylthiazol-2-yl]-2,5-diphenyl-tetrazolium bromide) reduction assay, as previously described [15]. Briefly, cells were seeded at 2 × 10^3^ cells per well in a 96-well plate and cultured overnight. The cells were then treated with different doses (5 µM, 10 µM, 15 µM, 20 µM, 25 µM, 30 µM, 35 µM, 40 µM in DMSO) for 72 h. Viability of the treated cultures was estimated by conversion of MTT to the insoluble formazan after 3h incubation at 37 °C and 5% CO_2_. The purple-colored formazan crystals were dissolved in DMSO, and absorbance was recorded at wavelengths 570/640 nm using the Bio-Rad Benchmark Plus Microplate Reader. The percent of viable cells was calculated OD_treated cells_/OD_control cell wells_ × 100.

### 5.5. Estimation of Total Reactive Oxygen Species (ROS)

The relative levels of intracellular ROS produced in untreated and drug-treated cells were analyzed by fluorimetry using the fluorescent probe H2DCFDA (Sigma-Aldrich, #D6883, St. Louis, MI, USA), as described previously [42,43]. Briefly, cells cultured in a 96-well plate at 1 × 10^4^ cells per well were treated with drugs of interest for 24 h rinsed with sterile PBS to remove excess drugs, and incubated with 10 μM DCFDA for 30 min at 37 °C and 5% CO_2_. Following three rinses with PBS, the fluorescence intensity was recorded at excitation: 485/20, emission: 528/20 using a BioTek Synergy HTX Multimode fluorescence plate reader (Agilent Technologies, Inc., Santa Clara, CA, USA), and the data were represented as percentage change compared to the respective untreated control.

### 5.6. Cell Cycle Phase Fractionation Study Through Flow Cytometry

Cells cultured in 35 mm diameter, 6-well cluster plates (Corning Inc., Corning, NY, USA) overnight at a density of 0.3 × 10^6^ cells/well, and treated with various compounds at selected concentrations for 24 h. After completion of treatment, cells were washed twice with PBS, followed by the addition of Propidium Iodide (PI) containing hypertonic solution [(0.1 mg/mL PI), 50mg Na-citrate, 15 µL of IGEPAL CA-630], 1 mL in each well, and incubated for 30–45 min at 37 °C and 5% CO_2_. After that data acquired using the NovoCyte Quanteon Flow Cytometer was analyzed using NovoExpress software (version 1.6.1) [43,44,45].

### 5.7. Assay to Measure Apoptosis Activity

We used a fluorescence-based Annexin V binding assay to determine the fraction of the cell population undergoing early and late apoptosis due to the cytotoxicity of the drugs [15]. Cells plated in a 60 mm dish overnight at 0.3 × 10^6^ cells/well were incubated with the selected drug at several doses for 24 h. Subsequently, treated cells were labeled with FITC-Annexin V using a kit (eBioscience™ Annexin V Apoptosis Detection Kits; Invitrogen # 88-8005-72, Thermo Fisher Scientific, Seoul, Korea) as per the manufacturer’s instructions. Intact cell suspension labeled with FITC-Annexin V and PI was run on the NovoCyte Quanteon flow cytometer, and data were acquired in list mode. Acquired data were analyzed using NovoExpress software (version 1.6.1). Cells treated with Staurosporine (0.2 µM) act as a positive control [43].

### 5.8. Biotinylated Pull-Down of Protein Followed by LC-MS/MS Analysis

The 5637 and MB49 cells were cultured and treated with higher concentrations of **5c** (50 µM) for short period of times (10 min & 60 min) followed by whole cell lysis (WCL) preparation of both treated and untreated cells using modified RIPA buffer [NaCl (100mM), TrisCl (20mM, pH 7.4), EDTA (10 mM), TritonX-100 (0.5%), Glycerol (5%), Protease inhibitor cocktail (1x)] with gentle continuous rotation. The protein content of each WCL was estimated using Pierce™ BCA Protein Assay kit (Thermo-Scientific # 23225). The 100 µL of resuspended Dynabeads™ MyOne™ Streptavidin C1 (Invitrogen # 65001) were washed with PBST (PBS with 0.01% Tween™20) in a magnet stand as recommended in the manufacturer’s instructions. The washed beads were incubated with 200 µg of each WCL in separate tubes at room temperature for 2 h in a gentle end-over-end mixing. After incubation, beads were washed 3–5 times with PBST (0.01% Tween-20) on a magnetic stand and beads were decanted by after spinning and magnetization. The samples were stored at −80 °C till further analysis using LC-MS/MS by the Proteomics and Mass Spectrometry Core Laboratory of Augusta University.

## Figures and Tables

**Table 1:** Antiproliferation properties of the UA-biotin conjugates **5a-d** and UA.

S. No	Compd.	IC_50_ Values in µM (Mean ± SD) (SI)		
		5637	T24	MCF-12A
1	**5a**	39.31 ± 21.17 (15.92)	14.57 ± 4.97 (42.96)	626.00 ± 34.69
2	**5b**	>10,000	107.7 ± 24.41 (0.68)	73.33 ± 0.12
3	**5c**	10.97 ± 0.19 (7.58)	14.20 ± 1.01 (5.85)	83.11 ± 0.30
4	**5d**	>10,000	581.50 (6.57)	3823.00
5	**1**	13.43 ± 0.35 (4.19)	17.52 ± 0.98 (3.21)	56.39 ± 7.60

**Table 2:** The IC_50_ concentration (μM) for each cell type was calculated using GraphPad Prism (v10.60.1) based on MTT results of 72 h exposure of UA and **5c**.

IC_50_ Concentrations (µM) ± SD (SI)			
S. No.	Cell Line	UA	5c
1	5637	13.43 ± 0.35 (4.99)	10.97 ± 0.19 (5.74)
2	HT-1376	21.9 ± 1.14 (3.06)	12.68 ± 0.40 (4.96)
3	T-24	17.52 ± 0.98 (3.82)	14.20 ± 1.01 (4.43)
4	MB49	10.71 ± 0.36 (6.26)	13.58 ± 0.18 (4.63)
5	UROtsa	67.01 ± 7.15	62.90 ± 3.84

**Table 3:** Proteomics data of **5c** from pulldown assays of the MB49 and 5637 cell lines.

S. No.	Description	5637		MB-49	
		Fold Change 10 min/Ctrl	Fold Change 1 h/Ctrl	Fold Change 10 min/Ctrl	Fold Change 1 h/Ctrl
1	ATP synthase subunit alpha	1.63	2	1.26	1.26
2	ATP synthase subunit beta	3.29	5	1.31	1.82
3	ATP synthase subunit O	3	3	4	5
4	ATP-binding cassette sub-family A	3	5	1	2
5	Coiled-coil domain-containing protein	0	2	0	0
6	D-3-phosphoglycerate dehydrogenase	15	10	0.8	1.6
7	DNA topoisomerase	0.08	0.11	1	1.75
8	Elongation factor 1-beta (EF1β)	2.35	2.35	5	5
9	Elongation factor 1-delta (EF1δ)	8	7	1.4	2
10	Elongation factor 1-gamma (EF1γ)	4	6	4	1
11	Elongation factor 2 (eEF2)	44	50	3	2
12	Eukaryotic initiation factor 4A (eIF4A)	22	19	1	3
13	Eukaryotic translation initiation factor 2 (eIF2)	14	7	2	0
14	Eukaryotic translation initiation factor 5B (eIF5B)	32	34	3	2.75
15	Filamin-A (FLNA)	16	18	3.33	4
16	HSC 71	5.25	6.25	1.5	2
17	HSP 90-alpha (HSP90α)	119	111	1.07	1.25
18	HSP 90-beta (HSP90β)	93	97	1.40	1.71
19	Heterogeneous nuclear ribonucleoprotein U (HNRNPU)	4.33	4.33	2.38	2.13
20	Poly(U)-binding-splicing factor 60 (PUF60)	6	5	2.33	3.33
21	Rab-like protein 6 (RABL6)	1.33	2.67	3	5.5
22	Ras GTPase-activating-like protein IQGAP1	3.5	2.25	1	3
23	Receptor of activated protein C kinase 1 (RACK1)	6	6	0.88	1.29
24	Ribosomal L1 domain-containing protein 1 (RSL1D1)	5	4	1.33	1.56
25	RNA-binding protein 39 (RBM39)	3.5	3	2	1.71
26	Splicing factor U2AF 65 kDa subunit (U2AF65)	14	10	4	3
27	T-complex protein 1 subunit theta	14	12	2	3
28	Histone H2A type 2-C (HIST2H2AC)	3.48	3.04	1.58	1.27
29	Histone H2A.Z	5.85	4.78	1.86	1.20
30	Histone H2B type 1-M	3.16	1.37	2.04	1.81
31	Histone H4	13.23	11.08	1.64	1.18

## Data Availability

Data are contained within the article or Supplementary Materials.

## Supplementary Materials

The following supporting information can be downloaded at: https://www.mdpi.com/article/10.3390/molecules30234588/s1, Table S1: The Cell cycle phase distribution of different cells after the treatment of UA, 5c for 24 h. Data shown here is the mean ± SD of n = 3; Figures S1–S24: IR, 1H-,13C-NMR, and HRMS spectra of all synthesized molecules.
