# Supplementary material for: Biotin-Linked Ursolic Acid Conjugates as Selective Anticancer Agents and Target-Identification Tools for Cancer Therapy
Source: Molecules. 2025 Nov 28;30(23):4588. doi: 10.3390/molecules30234588 (PMC12692861; doi:10.3390/molecules30234588)
Supplement: Supplementary file 1 [file molecules-30-04588-s001.zip › molecules-3990853-supplementary.pdf]

Supporting Information

Biotin-Linked Ursolic Acid Conjugates as Selective Anticancer Agents and Target-Identification Tools for Cancer Therapy

Riham M. Bokhtia [1,†], Kunj Bihari Gupta [2,†], Annabella Natalini [3], Theerth Vikas Srinivasan [3,4], Nihal Amineni [3], Sophia Ying [3], Rajeev Shakuja [5,6], Guido F. Verbeck [3], Bal L. Lokeshwar [2,*] and Siva S. Panda [3,7,*]

[1] Department of Pharmaceutical Organic Chemistry, Faculty of Pharmacy, Zagazig University, Zagazig 44519, Egypt
[2] Georgia Cancer Center, Augusta University, Augusta, GA 30912, USA
[3] Department of Chemistry and Biochemistry, Augusta University, Augusta, GA 30912, USA
[4] Department of Biomedical Engineering, Georgia Institute of Technology, Atlanta, GA 30332, USA
[5] Department of Chemistry, University of Delhi, New Delhi, Delhi 110007, India
[6] Department of Chemistry, Birla Institute of Technology and Science, Pilani, Rajasthan 333031, India
[7] Department of Biochemistry and Molecular Biology, Augusta University, Augusta, GA 30912, USA
* Correspondence: blokeshwar@augusta.edu (B.L.L.); sipanda@augusta.edu (S.S.P.)
† These authors contributed equally to this work.

Table S1. The Cell cycle phase distribution of different cells after the treatment of UA, **5c** for 24h. Data shown here is the mean ± SD of n=3

Cell Line	Treatment	Cell-Cycle Phase-Fractions (% of total)		
		G1	S	G2/M
**5637**	**Ctrl**	39.44 ± 1.11	42.95 ± 1.90	17.61 ± 0.79
	**UA**	43.52 ± 0.88	37.89 ± 1.16	18.59 ± 2.05
	**5c**	47.78 ± 0.60	35.42 ± 1.33	16.79 ± 0.73
**HT-1376**	**Ctrl**	43.31 ± 0.73	40.21 ± 0.36	16.48 ± 0.37
	**UA**	52.40 ± 0.56	30.43 ± 0.48	17.17 ± 1.04
	**5c**	52.11 ± 0.06	31.73 ± 0.65	16.15 ± 0.71
**T24**	**Ctrl**	44.17 ± 0.13	35.38 ± 0.35	20.45 ± 0.48
	**UA**	57.76 ± 2.60	22.29 ± 0.77	19.95 ± 1.82
	**5c**	55.10 ± 0.37	29.70 ± 0.39	15.20 ± 0.02
**MB49**	**Ctrl**	47.29 ± 0.31	33.93 ± 1.46	18.78 ± 1.78
	**UA**	59.75 ± 0.77	21.13 ± 1.49	19.12 ± 1.09
	**5c**	53.52 ± 1.25	28.05 ± 0.56	18.43 ± 0.69

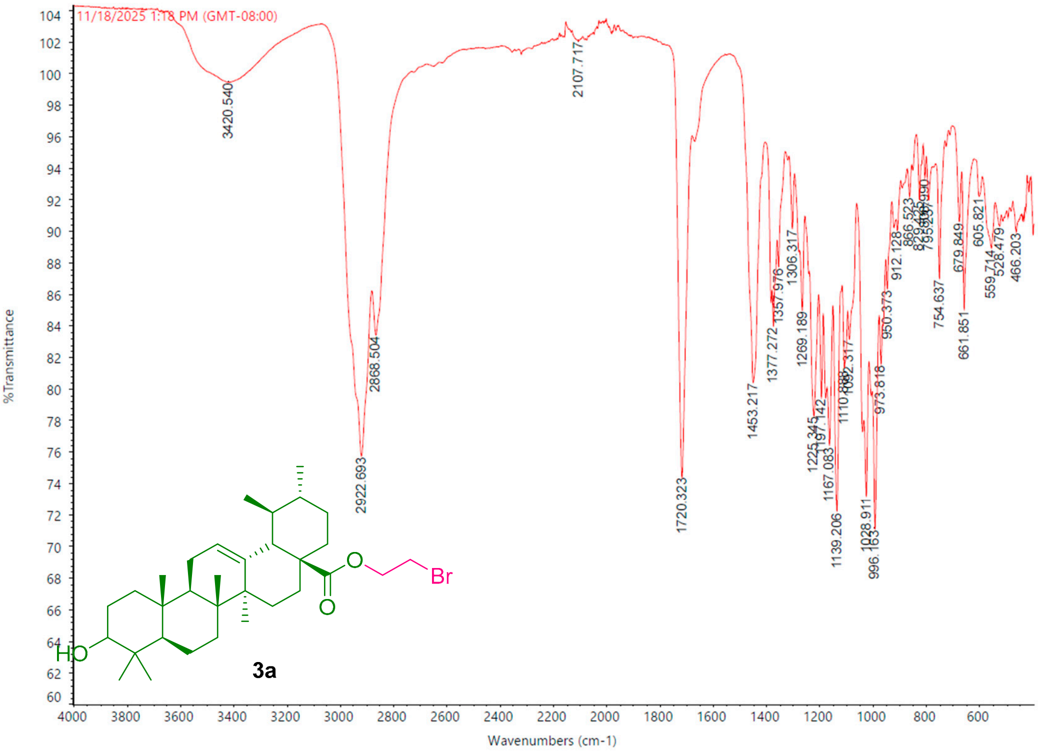

Figure S1. IR spectra of **3a**

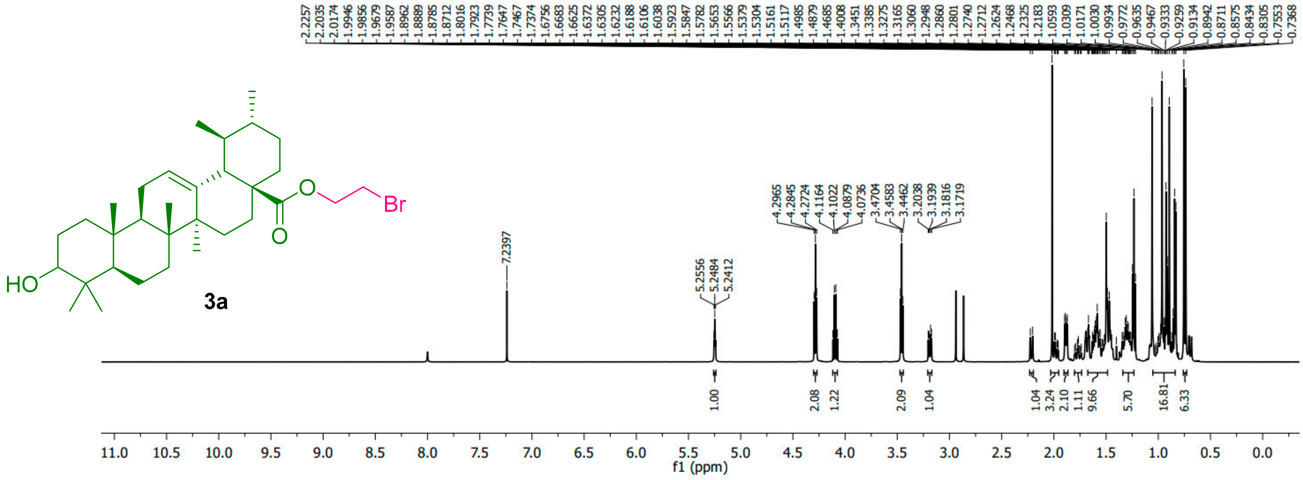

Figure S2. 1H NMR spectra of **3a**

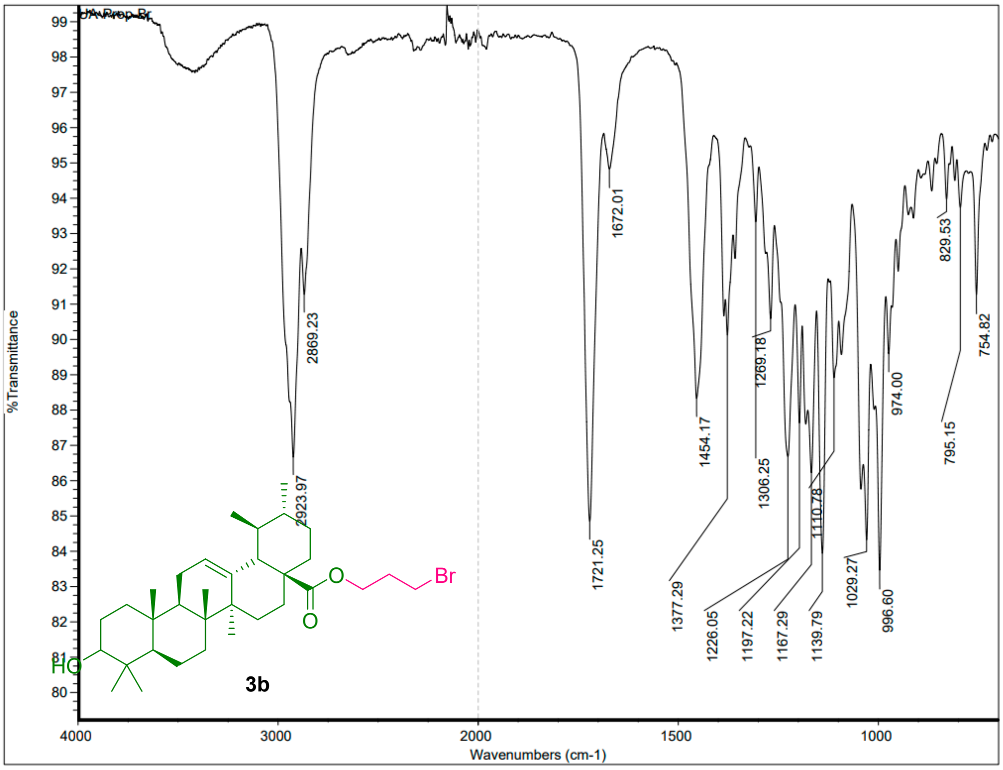

Figure S3. IR spectra of **3b**

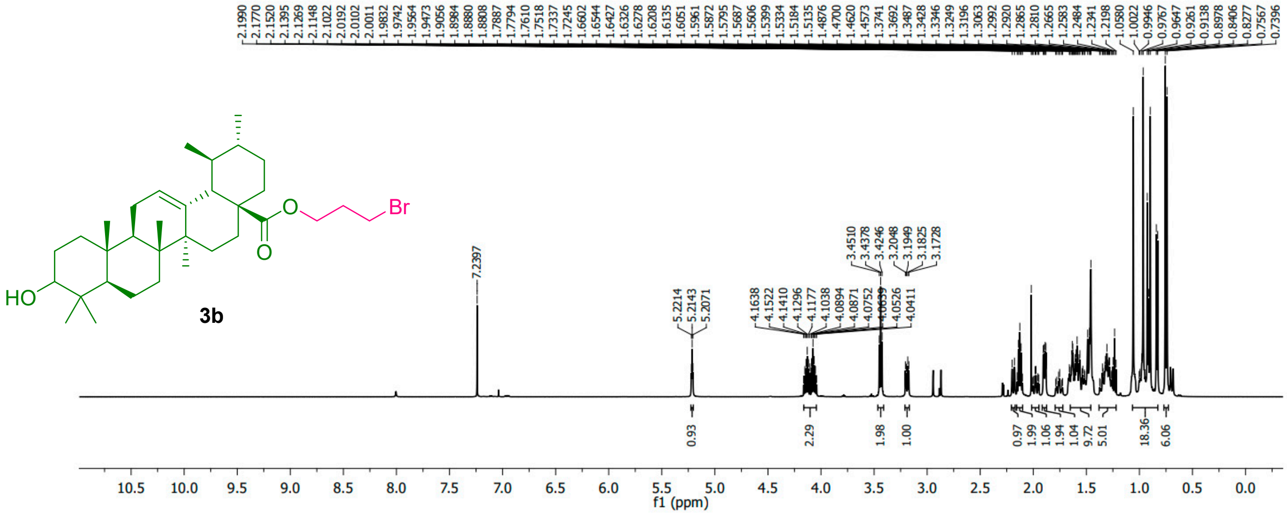

Figure S4. 1H NMR spectra of **3b**

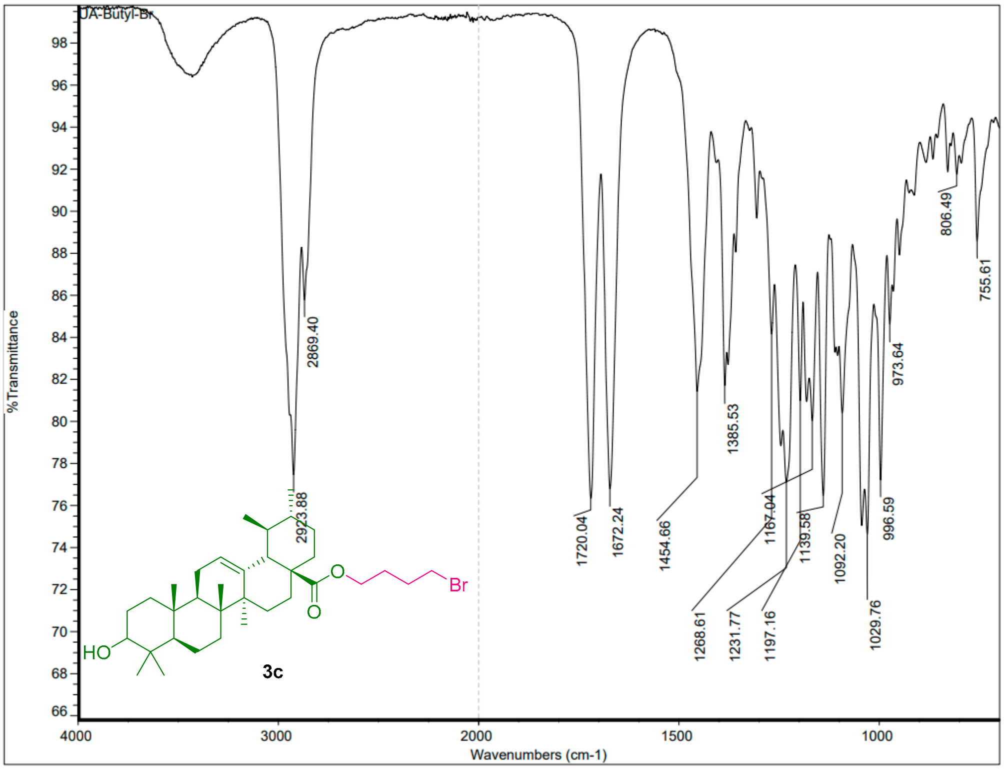

Figure S5. IR spectra of **3c**

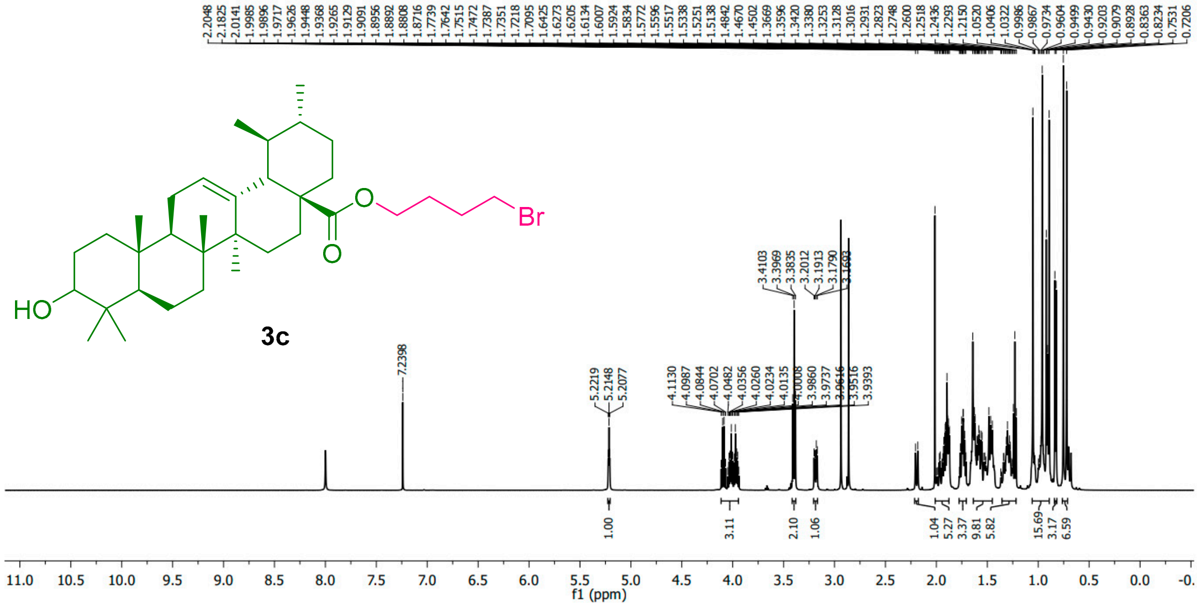

Figure S6. 1H NMR spectra of **3c**

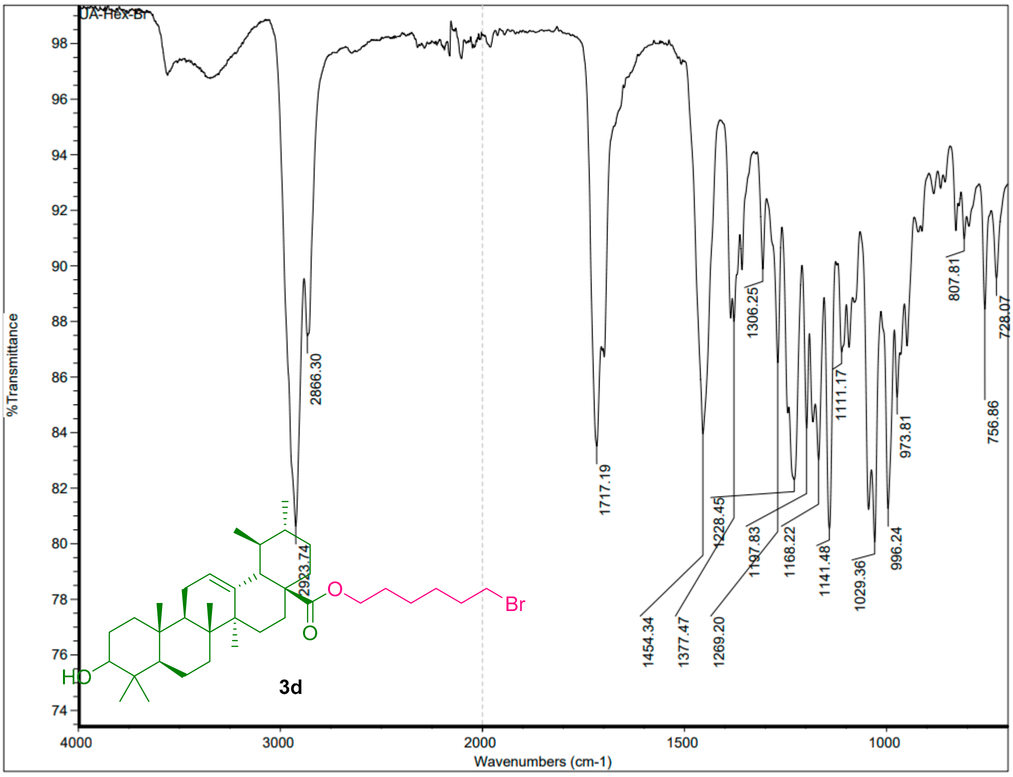

Figure S7. IR spectra of **3d**

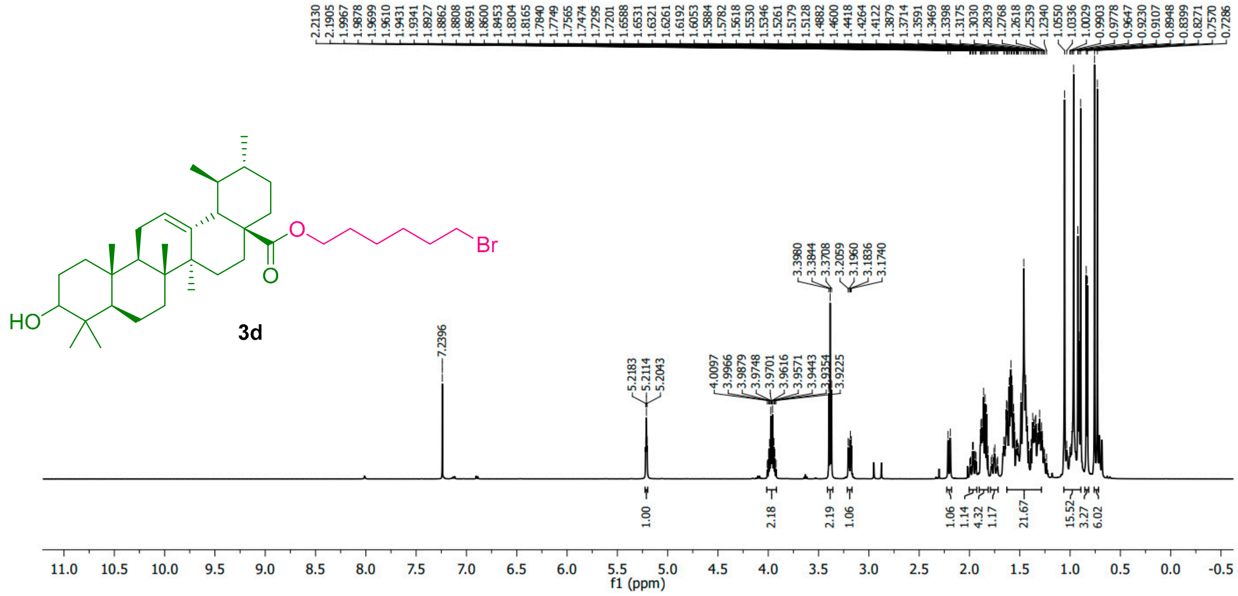

Figure S8. 1H NMR spectra of **3d**

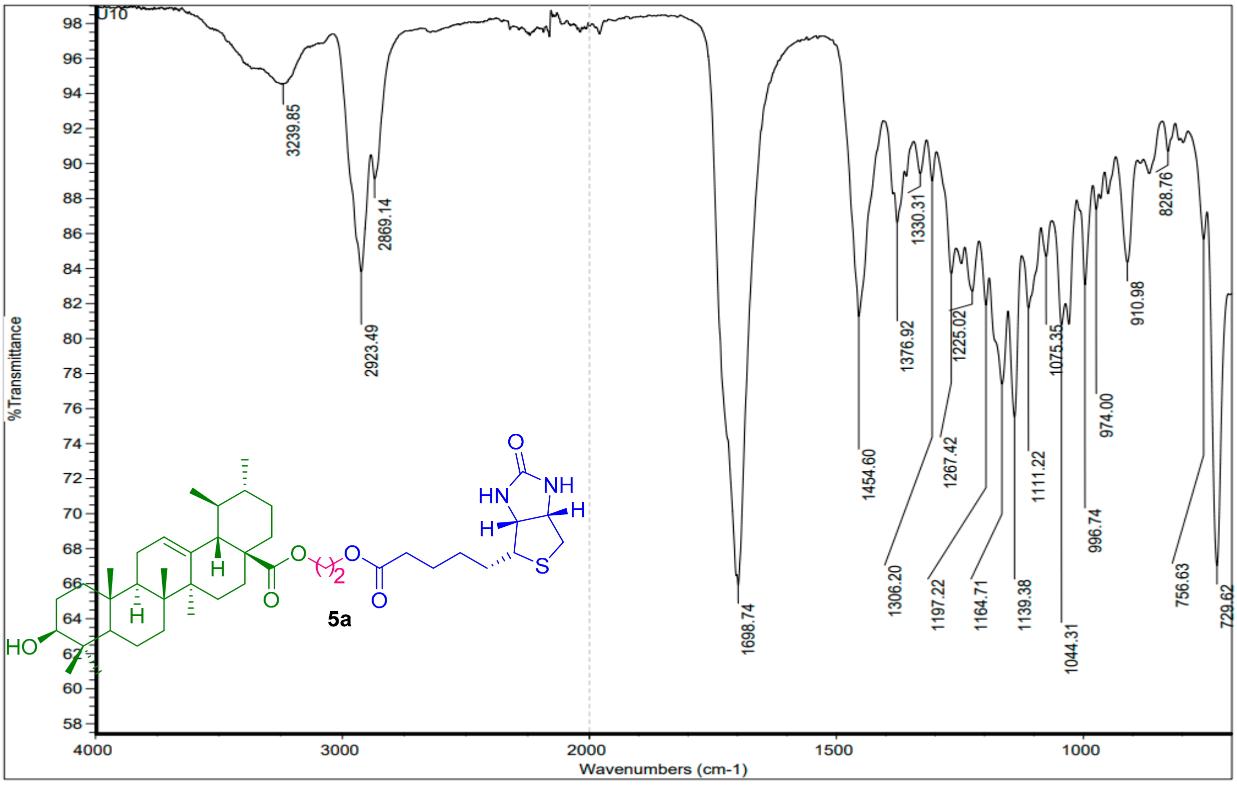

Figure S9. IR spectra of **5a**

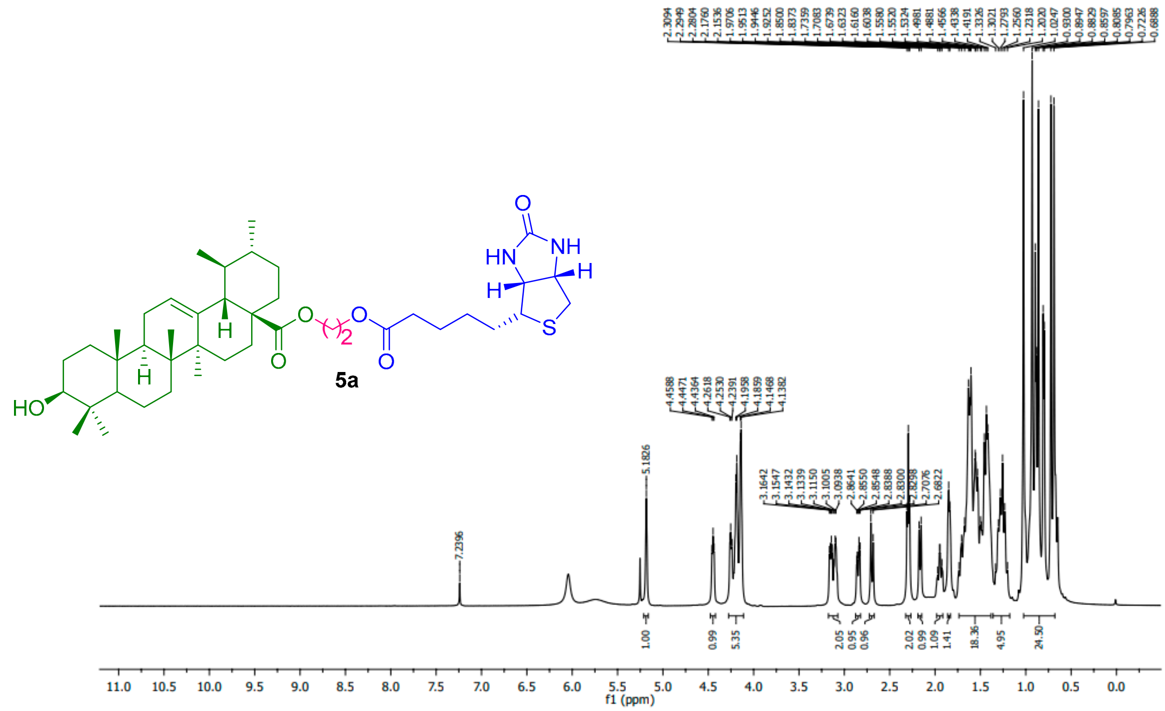

Figure S10. 1H NMR spectra of **5a**

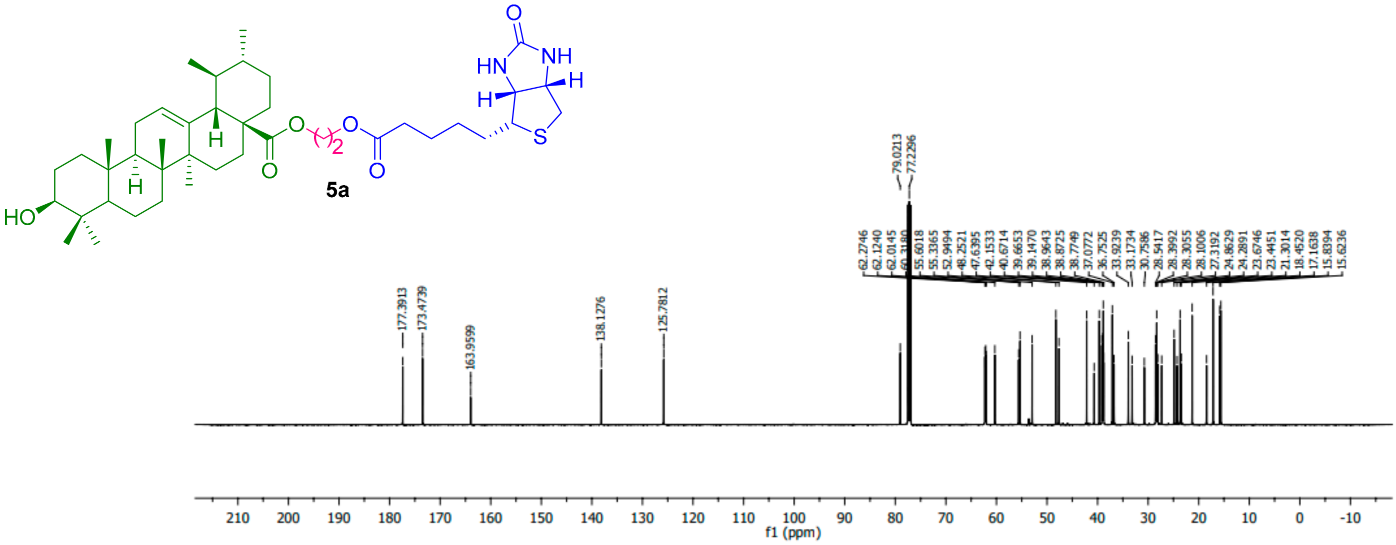

Figure S11. 13C NMR spectra of **5a**

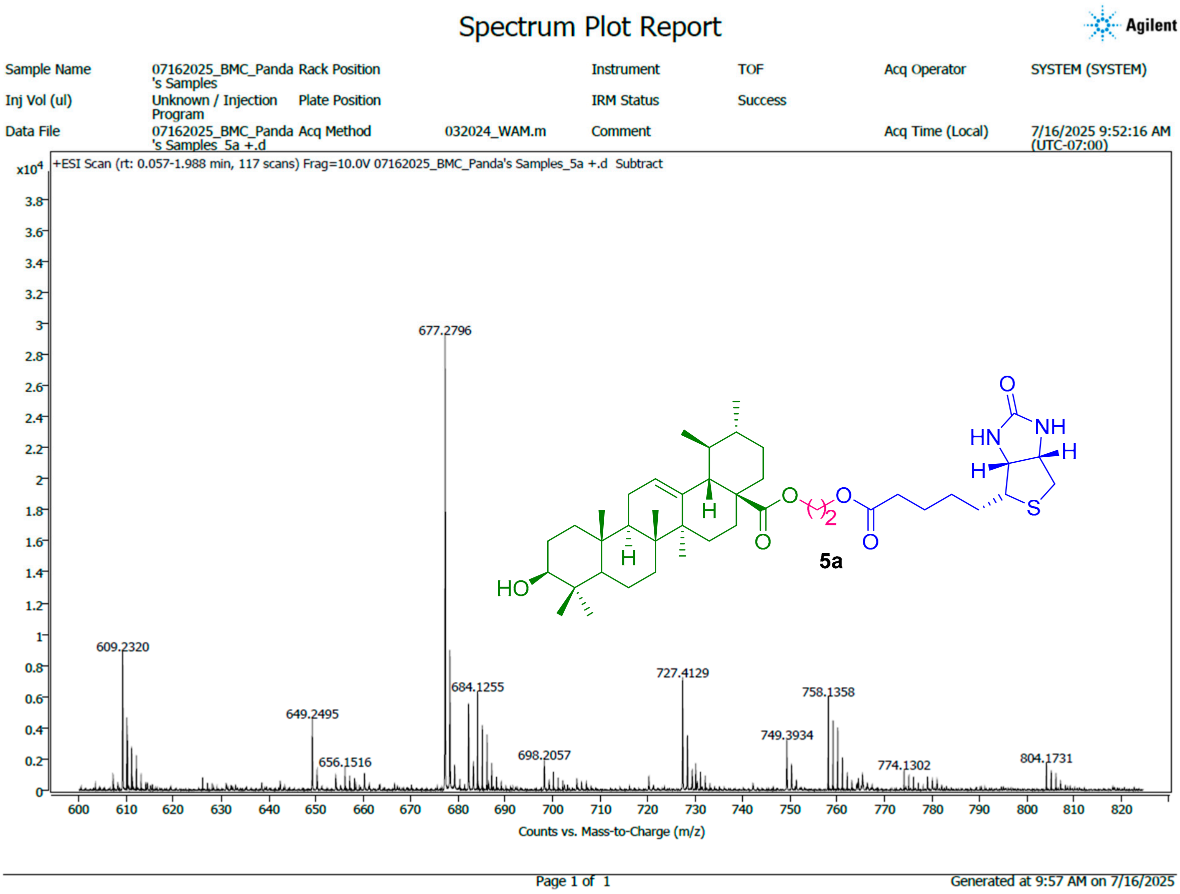

Figure S12. HRMS spectra of **5a**

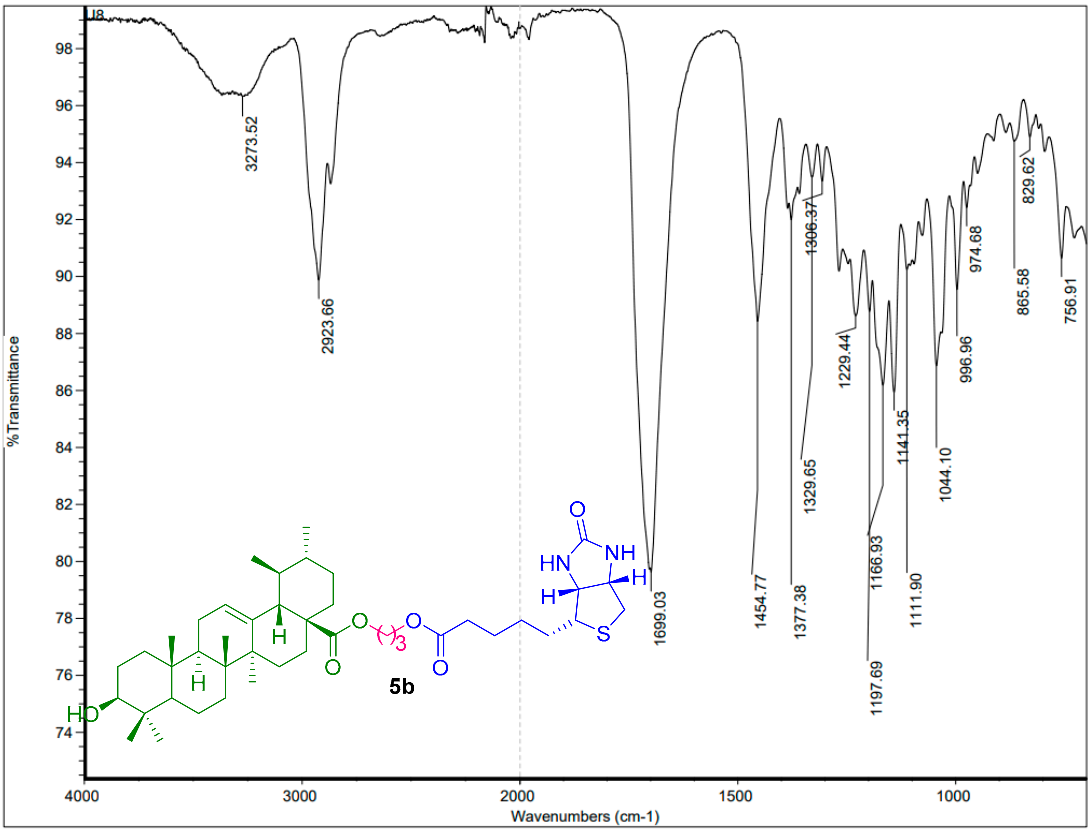

Figure S13. IR spectra of **5b**

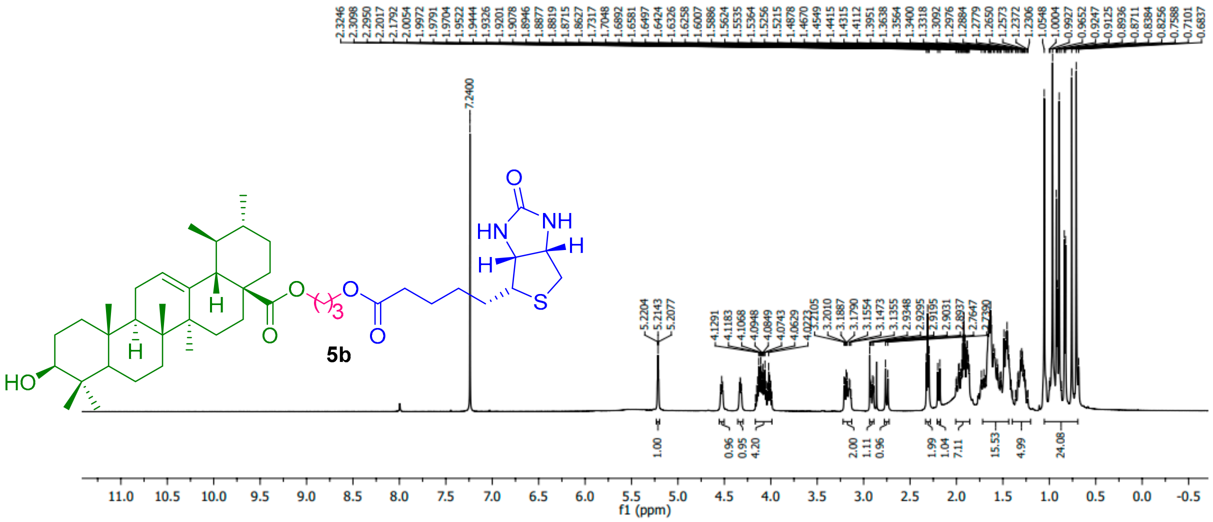

Figure S14. 1H NMR spectra of **5b**

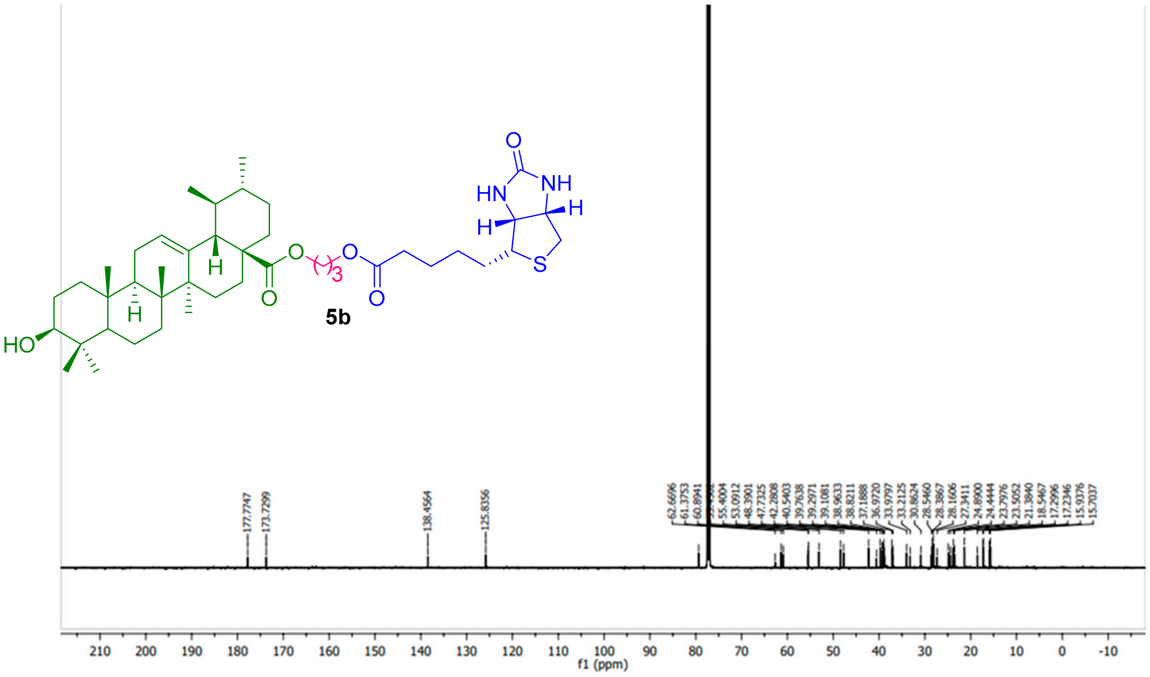

Figure S15. 13C NMR spectra of **5b**

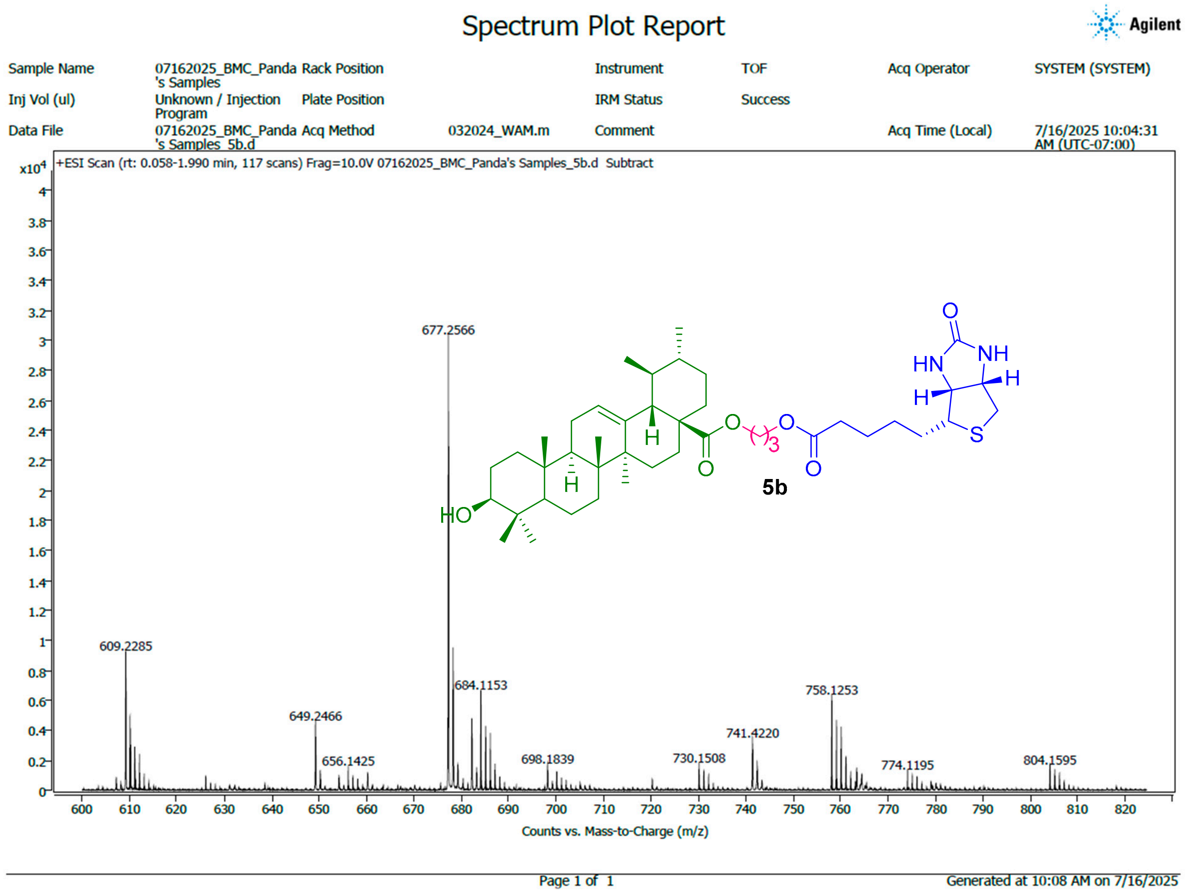

Figure S16. HRMS spectra of **5b**

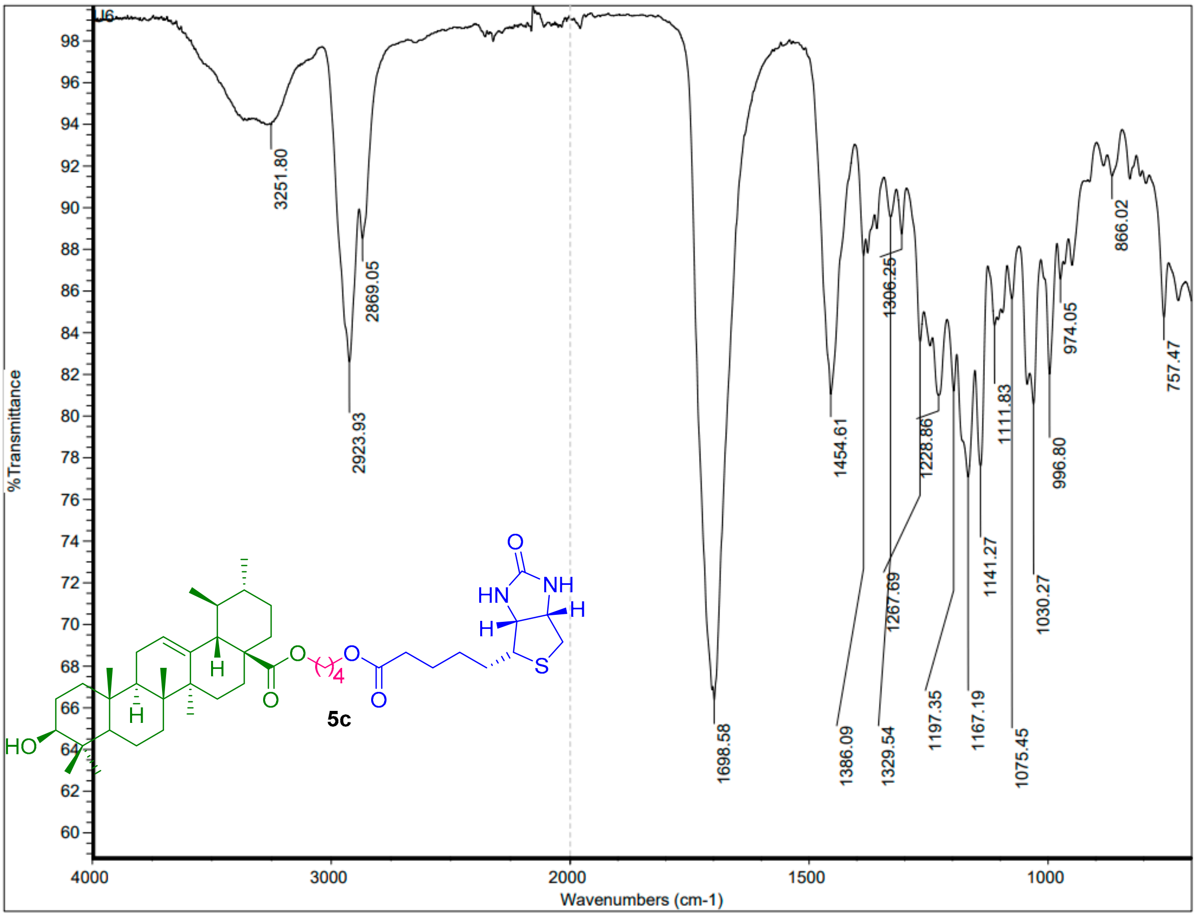

Figure S17. IR spectra of **5c**

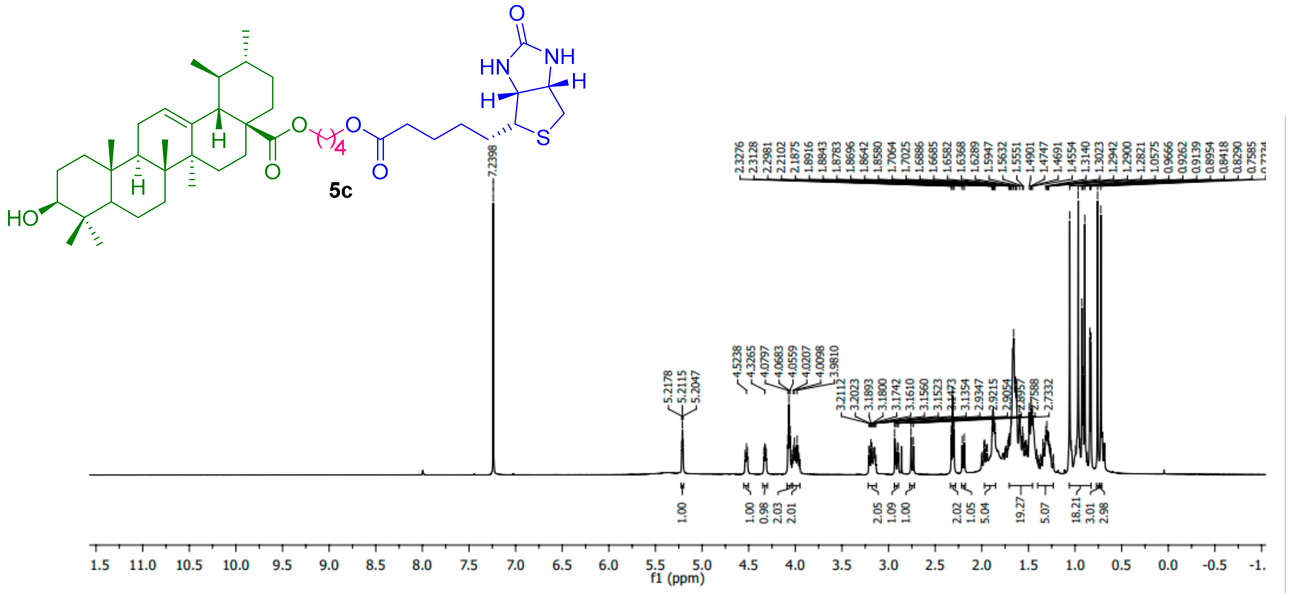

Figure S18. 1H NMR spectra of **5c**

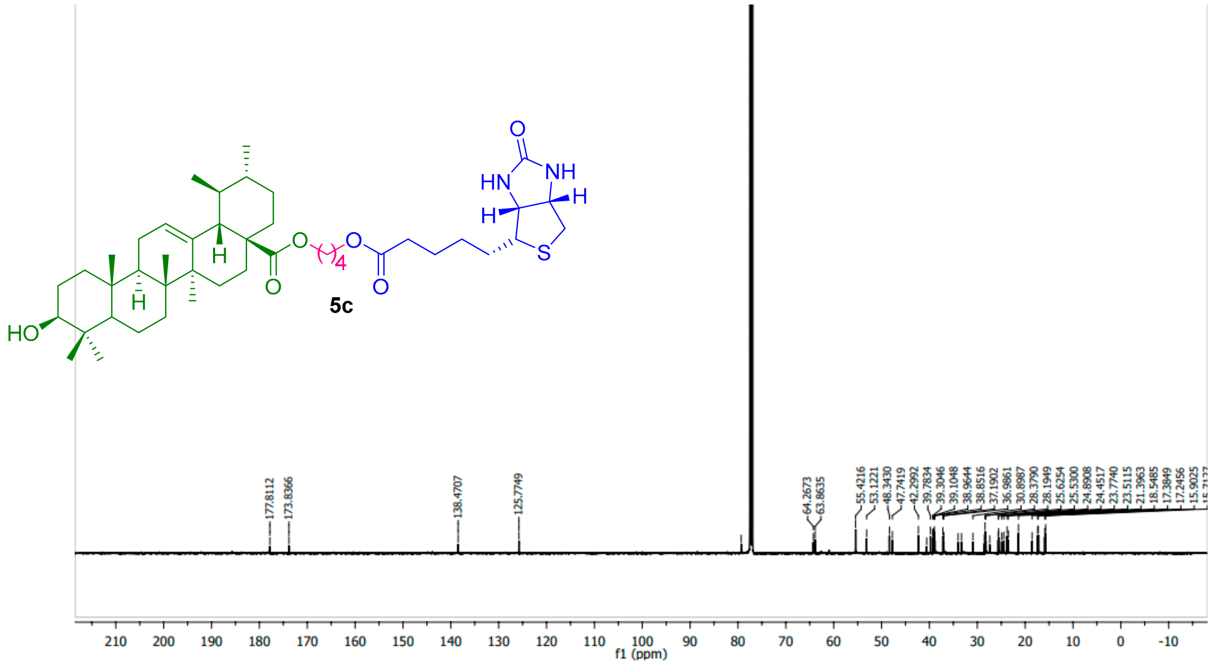

Figure S19. 13C NMR spectra of **5c**

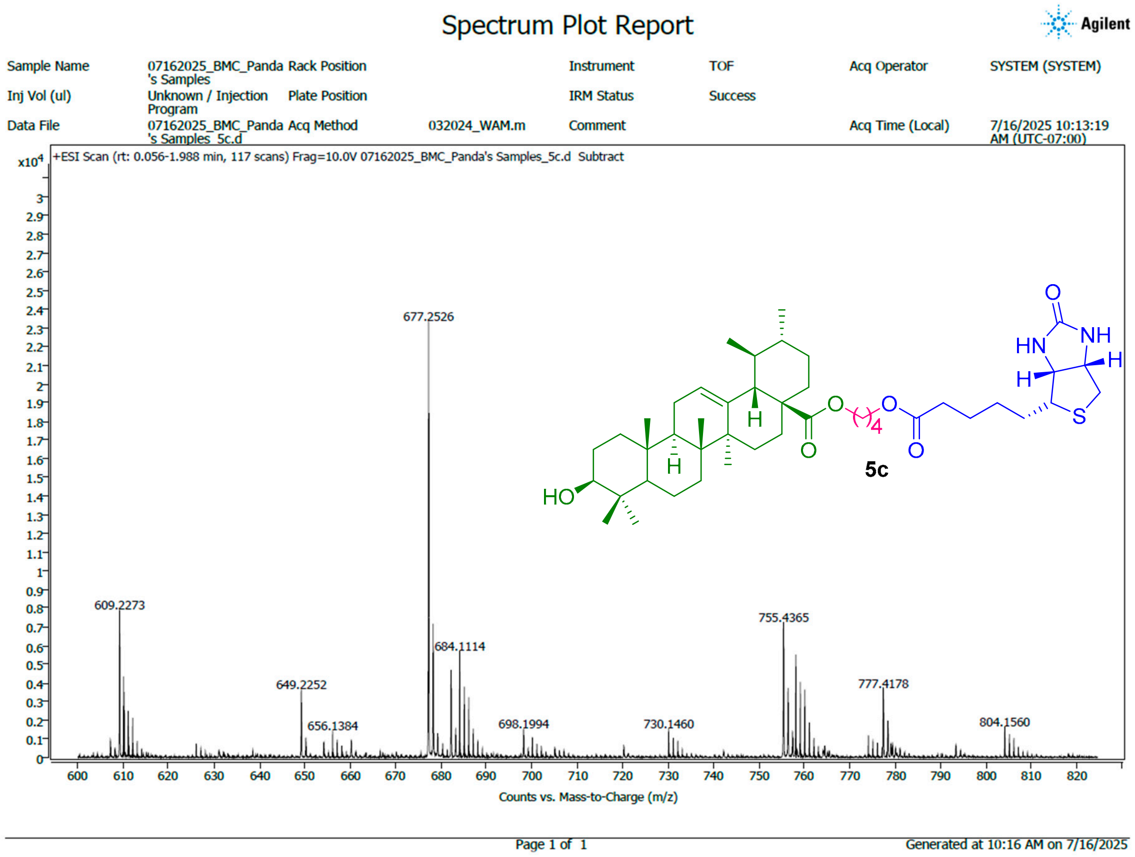

Figure S20. HRMS spectra of **5c**

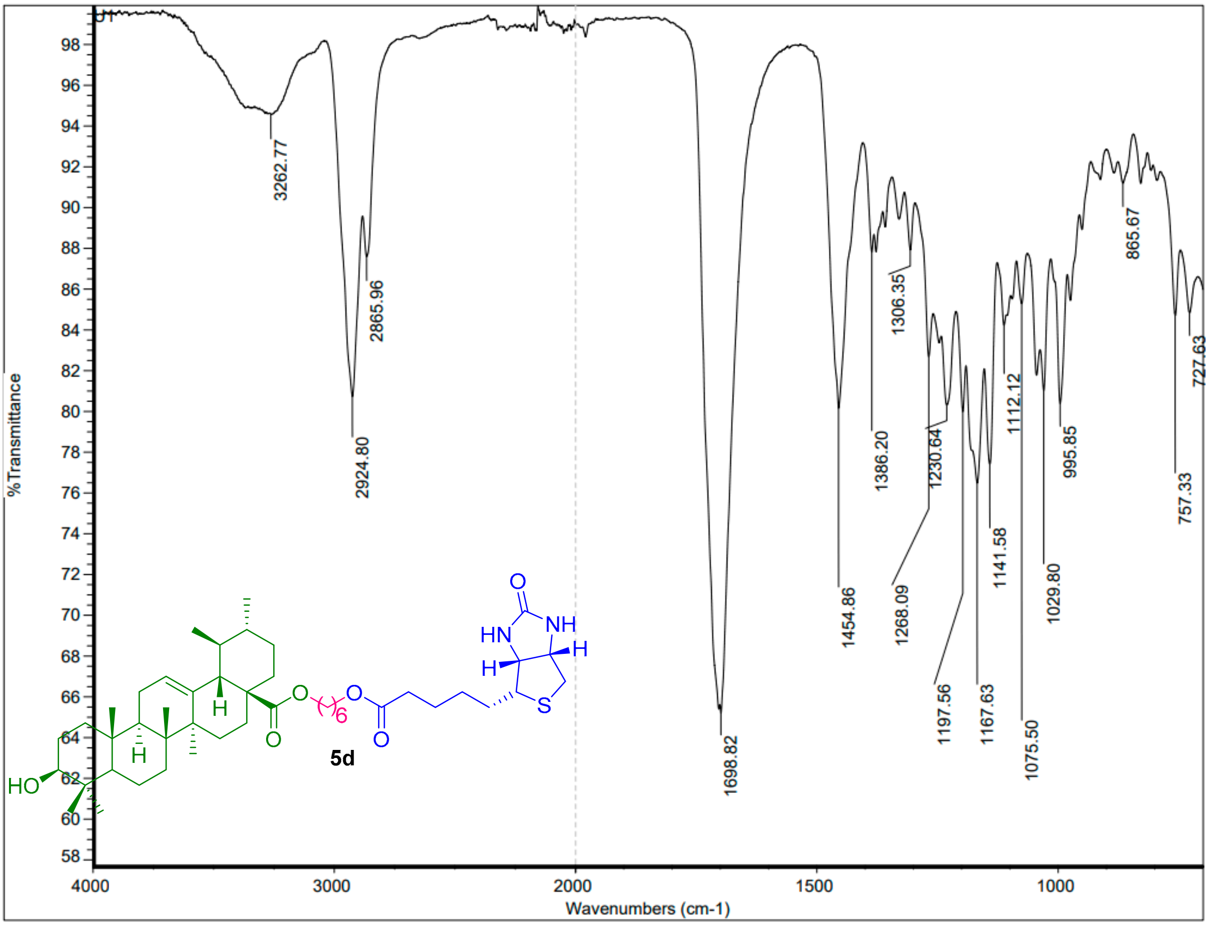

Figure S21. IR spectra of **5d**

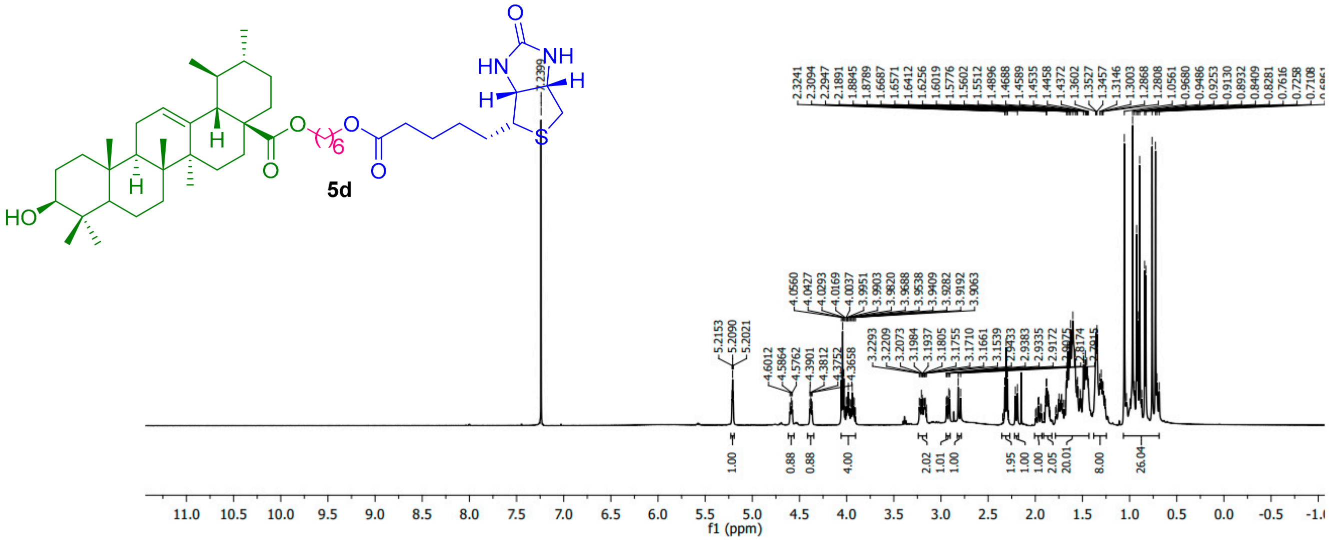

Figure S22. 1H NMR spectra of **5d**

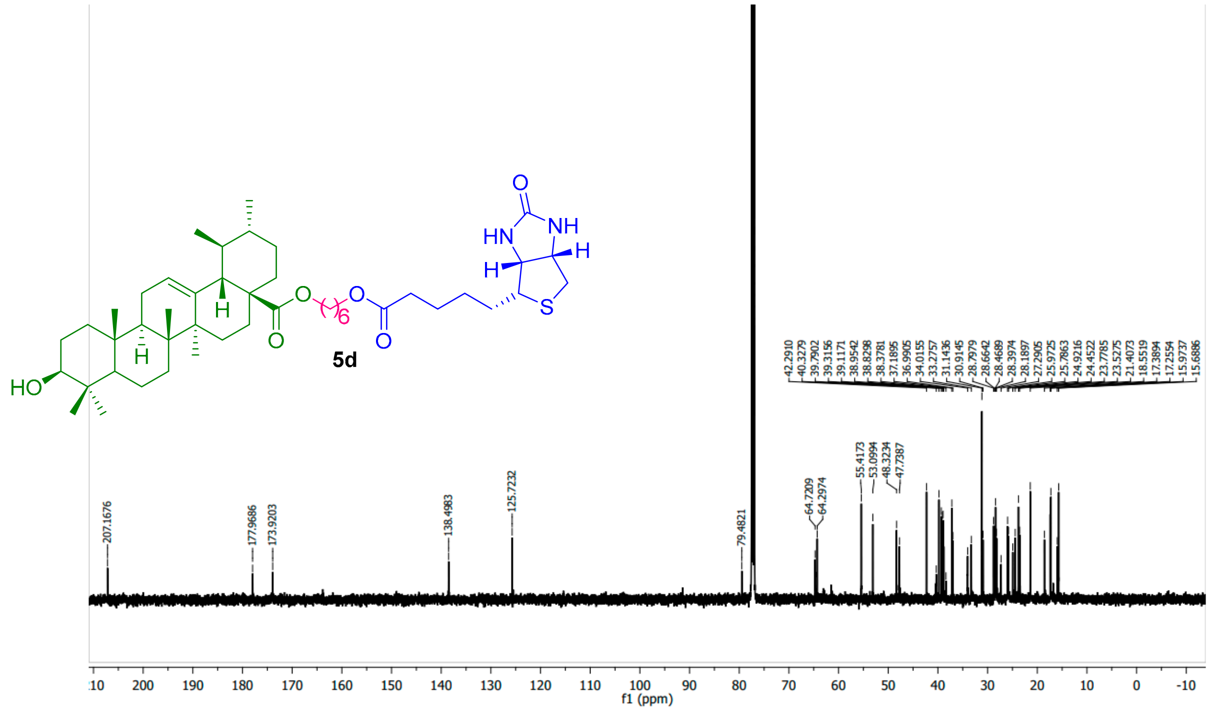

Figure S23. 13C NMR spectra of **5d**

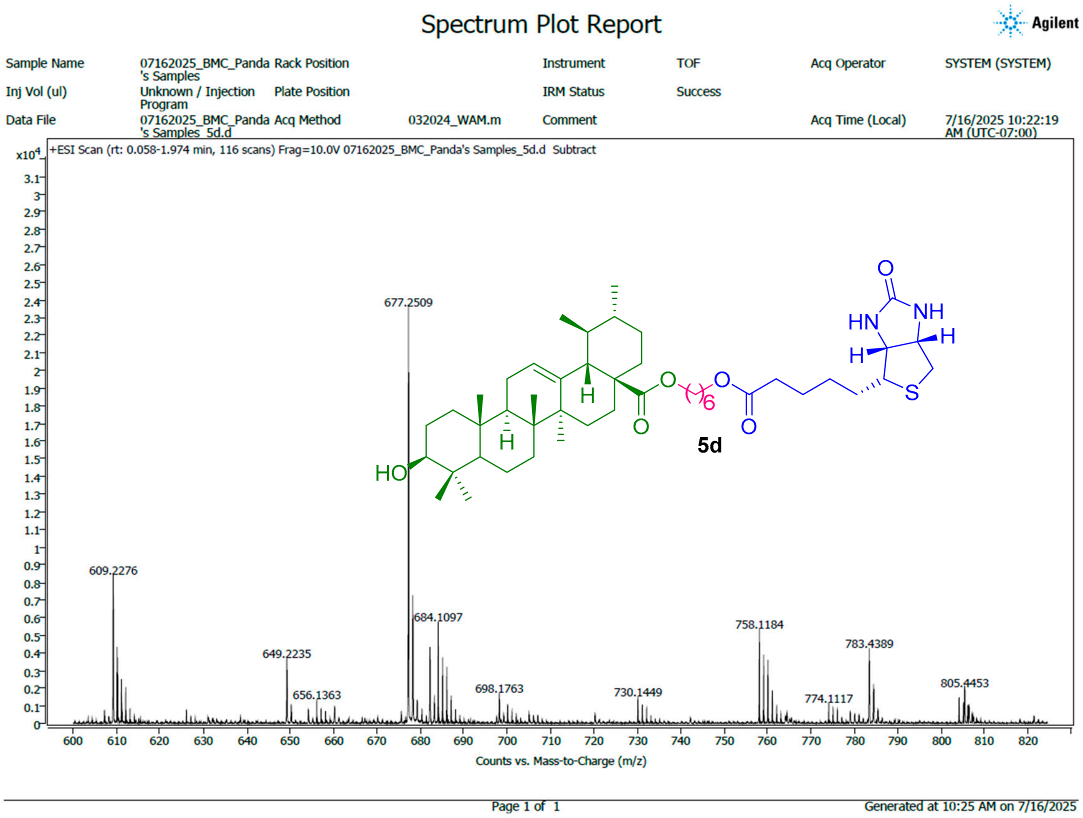

Figure S24. HRMS spectra of **5d**
